# Genome Sequence and Transcriptome Analyses of *Chrysochromulina tobin*: Metabolic Tools for Enhanced Algal Fitness in the Prominent Order Prymnesiales (Haptophyceae)

**DOI:** 10.1371/journal.pgen.1005469

**Published:** 2015-09-23

**Authors:** Blake T. Hovde, Chloe R. Deodato, Heather M. Hunsperger, Scott A. Ryken, Will Yost, Ramesh K. Jha, Johnathan Patterson, Raymond J. Monnat, Steven B. Barlow, Shawn R. Starkenburg, Rose Ann Cattolico

**Affiliations:** 1 Department of Genome Sciences, University of Washington, Seattle, Washington, United States of America; 2 Department of Biology, University of Washington, Seattle, Washington, United States of America; 3 Los Alamos National Laboratory, Los Alamos, New Mexico, United States of America; 4 University of Washington, Department of Pathology, Seattle, Washington, United States of America; 5 Electron Microscope Facility, San Diego State University, San Diego, California, United States of America; MicroTrek Incorporated, UNITED STATES

## Abstract

Haptophytes are recognized as seminal players in aquatic ecosystem function. These algae are important in global carbon sequestration, form destructive harmful blooms, and given their rich fatty acid content, serve as a highly nutritive food source to a broad range of eco-cohorts. Haptophyte dominance in both fresh and marine waters is supported by the mixotrophic nature of many taxa. Despite their importance the nuclear genome sequence of only one haptophyte, *Emiliania huxleyi* (Isochrysidales), is available. Here we report the draft genome sequence of *Chrysochromulina tobin* (Prymnesiales), and transcriptome data collected at seven time points over a 24-hour light/dark cycle. The nuclear genome of *C*. *tobin* is small (59 Mb), compact (∼40% of the genome is protein coding) and encodes approximately 16,777 genes. Genes important to fatty acid synthesis, modification, and catabolism show distinct patterns of expression when monitored over the circadian photoperiod. The *C*. *tobin* genome harbors the first hybrid polyketide synthase/non-ribosomal peptide synthase gene complex reported for an algal species, and encodes potential anti-microbial peptides and proteins involved in multidrug and toxic compound extrusion. A new haptophyte xanthorhodopsin was also identified, together with two “red” RuBisCO activases that are shared across many algal lineages. The *Chrysochromulina tobin* genome sequence provides new information on the evolutionary history, ecology and economic importance of haptophytes.

## Author Summary

Microalgae are important contributors to global ecological balance, and process nearly half of the world’s carbon each year. Additionally, these organisms are deeply rooted in the earths’ evolutionary history. To better understand why algae are such strong survivors in aquatic environments and to better understand their contribution to global ecology, we sequenced the genome of a microalga that is abundant in both fresh and salt water environments, but poorly represented by current genomic information. We identify protein-coding genes responsible for the synthesis of potential toxins as well as those that produce antibiotics, and describe gene products that enhanced the ability of the alga to use light energy. We observed that a day-night cycle, similar to that found in natural environments, significantly impacts the expression of algal genes whose products are responsible for synthesizing fats—a rich source of nutrition for many other organisms.

## Introduction

The contribution of photosynthetic algae to the maintenance of global ecological health is well recognized [1,2]. As primary producers, microalgal species support the survival of organisms at every trophic level. Additionally, they serve as important contributors to the earth’s geochemical cycles. Remarkably, ‘omics’ level characterization of algae is sorely lacking when compared to organisms related to human health or plant biotechnology [3].

Among algae, haptophytes represent an ancient and diverse lineage of eukaryotes. Estimates suggest that photosynthetic members of this taxon were present by the Neoproterozoic era (1000–520 Ma) [4]. The impact of haptophytes on global ecology (i.e., CO_2_ sequestration [5] and the production of dimethyl sulfide [6]), toxicity (i.e., polyketide products [7]); foam and mucilage production [8]; and their trophic value, given their high levels of fatty acids, has long been recognized [9]. However, metagenomic studies have only recently revealed the true extent of haptophyte dominance in aquatic ecosystems [10,11]. Data suggest that these algae may contribute “30 to 40% of the total photosynthetic standing stock in the world’s oceans” [12], and studies show previously unrecognized dominance in fresh water lakes [13]. The fact that some haptophyte species are mixotrophic as well as photosynthetic most likely provides a fitness versatility that helps explain the prevalence of these organisms within algal populations [14].

**Fig 1:**
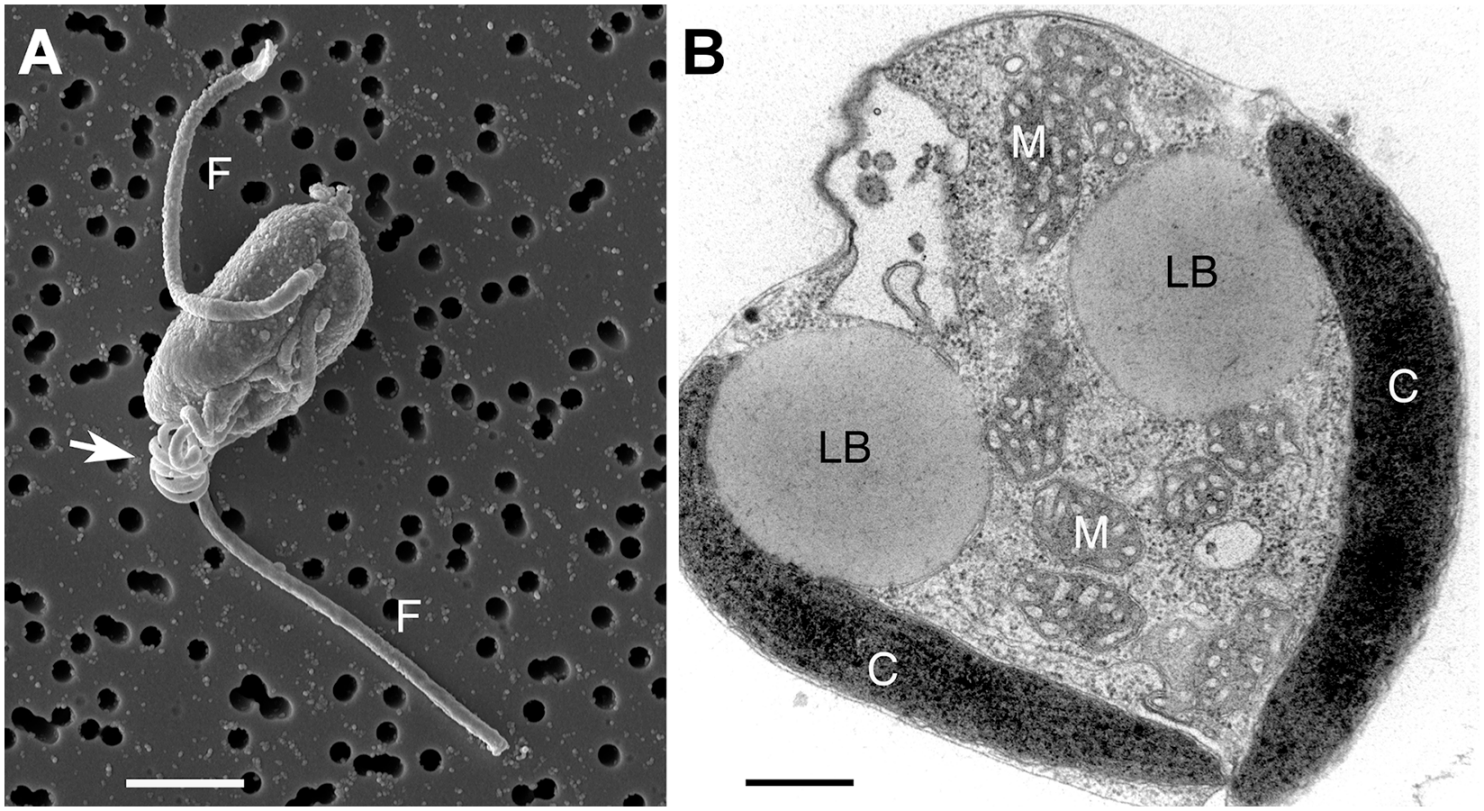
*Chrysochromulina tobin* cell structure. (A) Scanning electron micrograph of *C*. *tobin*. Two flagella are visible (marked F) along with the prominent coiled haptonema (white arrow). Scale bar represents 2.5 microns. (B) Electron micrograph of whole cell: Lipid body (LB); Mitochondrion (M); Chloroplast (C). Scale bar represents 500 nanometers.

Two subclasses of haptophytes are recognized [15]—the Pavlovophycidae (single clade) and the Prymnesiophycidae (encompassing 5 clades). *Emiliania huxleyi* (Isochrysidales) represents the only haptophyte for which a nuclear genome has been published to date [16]. This alga is a marine species well known for its calcified scales [17] and forms large blooms that are visible from space [18]. Genomic studies of the *E*. *huxleyi* pan-genome show significant genome variability among strains [19]. The haptophyte characterized in this study is *Chrysochromulina tobin* (Prymnesiophycidae: B2 clade). Members of the B2 clade to which this alga is associated have been shown to dominate some marine and freshwater ecosystems. For example, data suggest that >55% of all haptophyte sequences in a Mediterranean sampling site and ∼30% in a Norwegian sampling site were of the B2 haptophyte group [11,20,21]. *C*. *tobin* (Fig 1) is halotolerant, living in fresh to brackish water [22], and is phagocytotic, using a long haptonema to acquire prey. Unlike many haptophytes that are embellished with either organic or calcium carbonate scales [23], this organism is naturally wall-less. Though small (∼4.0 μm), 40% of its dry weight is lipid, with most fatty acids stored in two large, well-defined lipid bodies.

This study provides insight into several previously unreported genetic characteristics of a haptophyte. The fatty acid production pathways and their potential relationship to changes in lipid body morphology are examined. Furthermore, we identify several novel genes including a polyketide synthase-nonribosomal complex; genes encoding MATE antimicrobial products; the bacterially sourced, laterally transferred genes that encode RuBisCO activase isoforms and a unique xanthorhodopsin. Genomic sequencing and annotation of a representative in the Prymnesiales B2 clade provides critical information to resolve elusive evolutionary relationships among the deeply-rooted haptophyte algal taxon [24,25]. Additionally, genomic knowledge of haptophytes will enhance commercial endeavors that target aquaculture feed stocks, nutraceuticals, plastics or biofuel production. Given that many haptophytes are also toxic, genomic information will support efforts to understand the fundamental metabolic processes associated with the genesis of harmful algal bloom events.

## Results and Discussion

### Genome sequencing, assembly and annotation

*Chrysochromulina tobin* was isolated from a freshwater source, monocultured and bacterial contaminants reduced using reiterative cell sorting by flow cytometry followed by sequential antibiotic treatment. Purified total genomic DNA was used to prepare libraries for 454 and Illumina sequencing. The resulting draft assembly consisted of 3,472 contigs having an average length of ∼17 kb (Table 1). A 59 Mb genome was assembled representing an average read depth of over 100x (see Materials and Methods). The final genome assembly was found to contain a complete genomic sequence of one commensal bacterium (*Sphingomonas* sp.), that was removed from the assembled genome.

**Table 1:** *Chrysochromulina tobin* genome statistics.

Assembled genome size	59 Mb
Sequencing coverage	111x
Assembled contigs	3,472
Average contig size	∼17kb
N50 / L50	24,114 bp / 798 contigs
Contigs > 75kb	13
GC content	63.4%

### Genome properties

#### Genome size and compactness

*Chrysochromulina tobin* has a small, gene dense, 59 Mb genome that encodes an estimated number of 16,777 genes (S1 Table). Each gene contains, on average, a single intron. Average gene length is 1,899 bp (S1 Fig). In total, 61.4% of predicted genes had BLAST homologs (See Materials and Methods for details). This identification rate is similar to *E*. *huxleyi* and genomes within the sister stramenopile algal lineages that have been sequenced to date (49–69%) [26]. The 16,777 predicted *C*. *tobin* gene count (40% of the genome is protein coding sequence) is significantly lower than the ∼38,000 genes predicted [16] for the 141 Mb genome of *E*. *huxleyi* (21.9% protein coding). Such large differences in gene complement are not unexpected. For example, sequenced stramenopile genomes vary widely among taxa in size, gene number and coding capacity (S2 Table).

#### Sexual cycle

Among unsequenced haptophytes, haploid genome sizes, estimated by flow cytometry, range from ∼117 Mb for *Phaeocystis antarctica* [27] to ∼230 Mb for *Prymnesium polylepis* [7]. Flow cytometric estimates of *C*. *tobin*’s DNA content indicate a ∼55 Mb genome, which closely corresponds to the size estimated for the draft genome assembly presented above. This relationship suggests that *C*. *tobin* is haploid. The presence of a full complement of meiosis-related genes implies that a transient diploid state likely occurs in this organism, though diploidy has never been observed. Homologues to meiosis-related genes are also found in *E*. *huxleyi*, which displays both haploid and diploid phases [28].

#### Lateral gene transfer

Analysis of the *C*. *tobin* genome indicates that lateral gene transfer played a role in re-engineering and augmenting genome function in this organism. Imported exotic genes appear to have either eukaryotic or bacterial sourcing, and target both *C*. *tobin* nuclear and chloroplast genomes. For example, a duplicated nuclear-encoded *por* gene that is indispensable for chlorophyll synthesis has a chlorophytic algal origin [29] while the ribosomal subunit *rpl36* [30], xanthorhodopsin and RuBisCO activase (presented in this work) appear to be of bacterial origin [31].

### Cell growth and maintenance: Photoperiod impact

**Fig 2:**
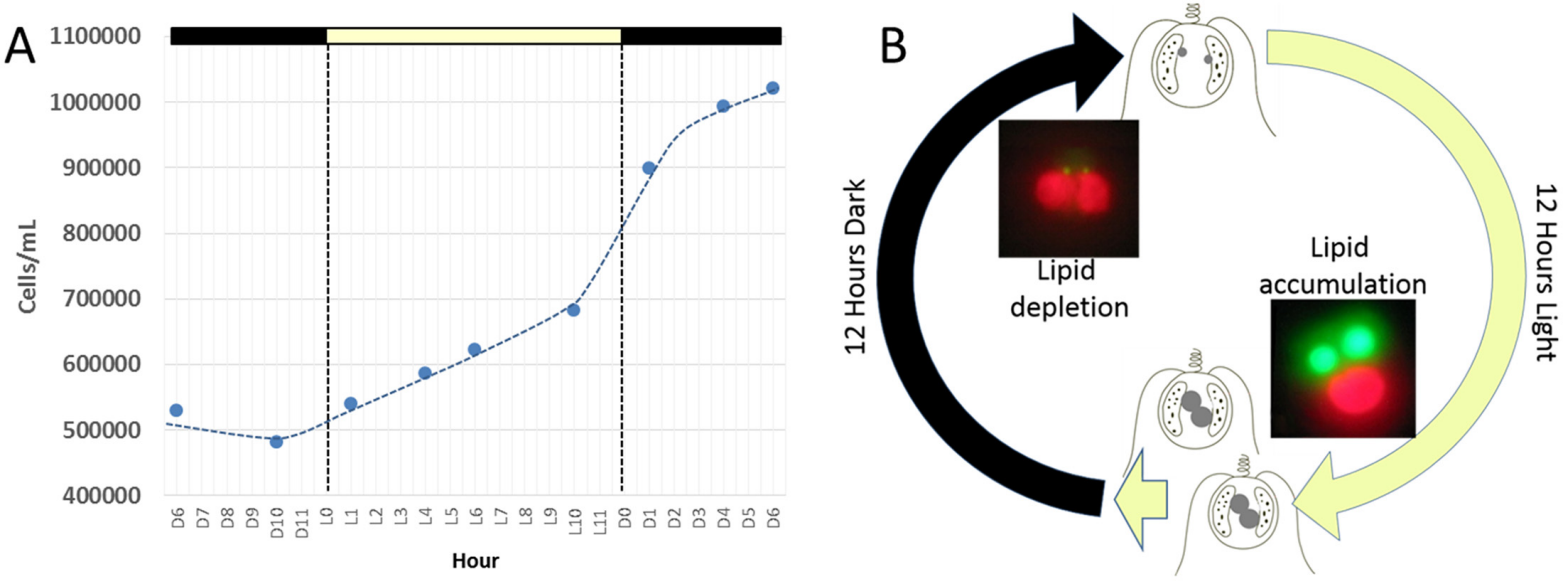
*Chrysochromulina tobin* displays photoperiod controlled cell division and lipid metabolism. (A) Cell division is observed to be highest during the light to dark transition. (B) Change in lipid body size is correlated to photoperiod when detected by BODIPY 505/515 dye incorporation (green); chloroplast auto-fluorescence (red).

Maintenance of *C*. *tobin* cultures depends on a light/dark photoperiod (continuous light leads to poor growth). A 12 hour light: 12 hour dark photoperiod partially synchronizes the culture (Fig 2A). Cell division initiates at approximately the tenth hour in the light (L10) and continues at a high rate with the onset of darkness, terminating approximately 6 hours in the dark (D6). Cultures double every 24 hours on a 12 hour light: 12 hour dark cycle in the logarithmic growth phase (Fig 2A). Most notably, during this biphasic 24 hour growth cycle, a dramatic change in the size of the lipid body is observed. The lipid bodies continuously increase in size during the light photoperiod, then shrink rapidly during the dark cycle (Fig 2B).

**Fig 3:**
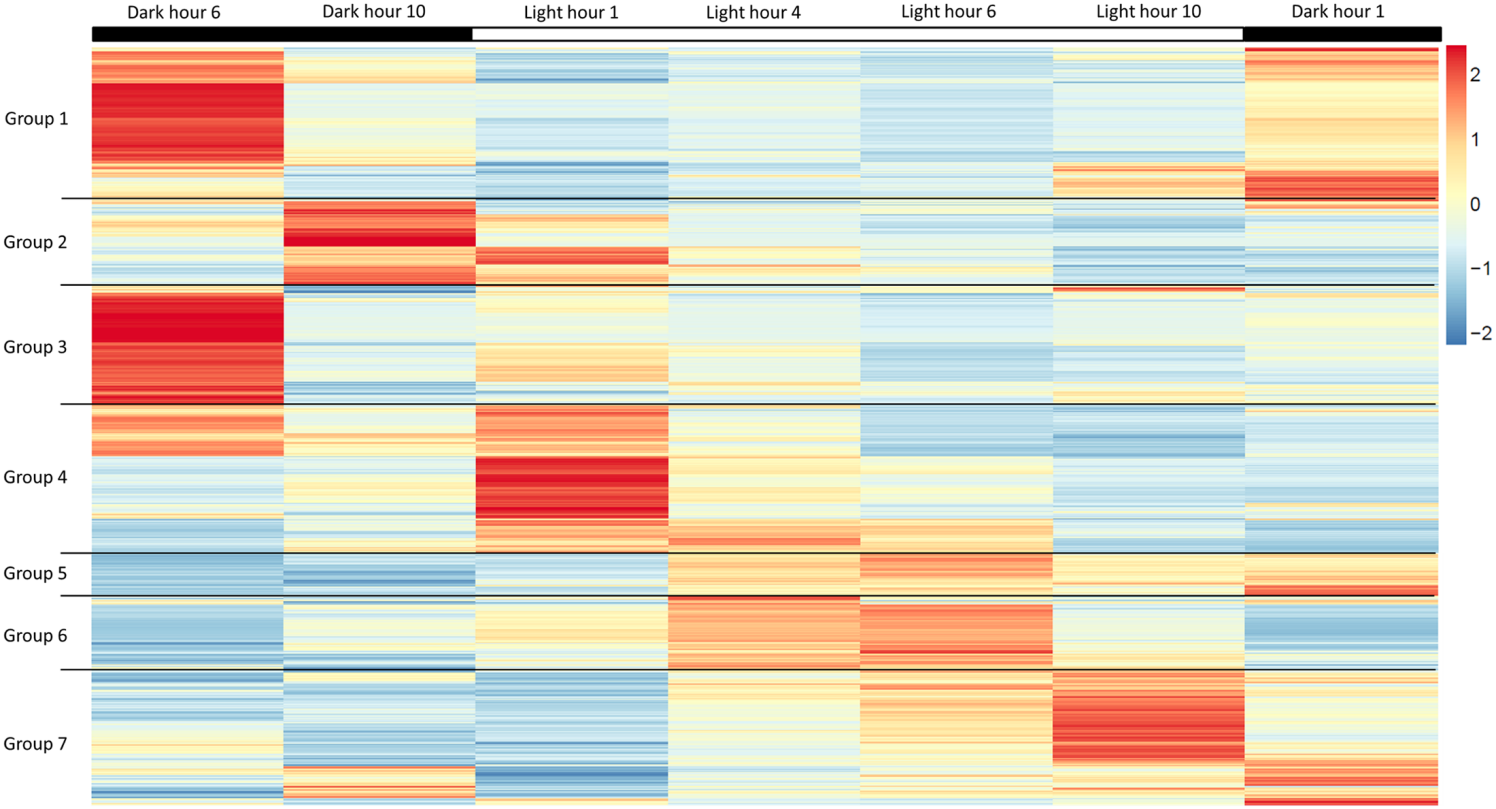
Heatmap of highly expressed genes over 7 time points during the 12 hour light: 12 hour dark photoperiod. Groups represent clusters of genes with similar expression patterns. A total of 1000 genes were included in this heatmap analysis. Color bar represents change in RNA abundance relative to average abundance of the 7 time point samples collected over 24 hours. Highest abundance in red and lowest abundance in blue.

**Fig 4:**
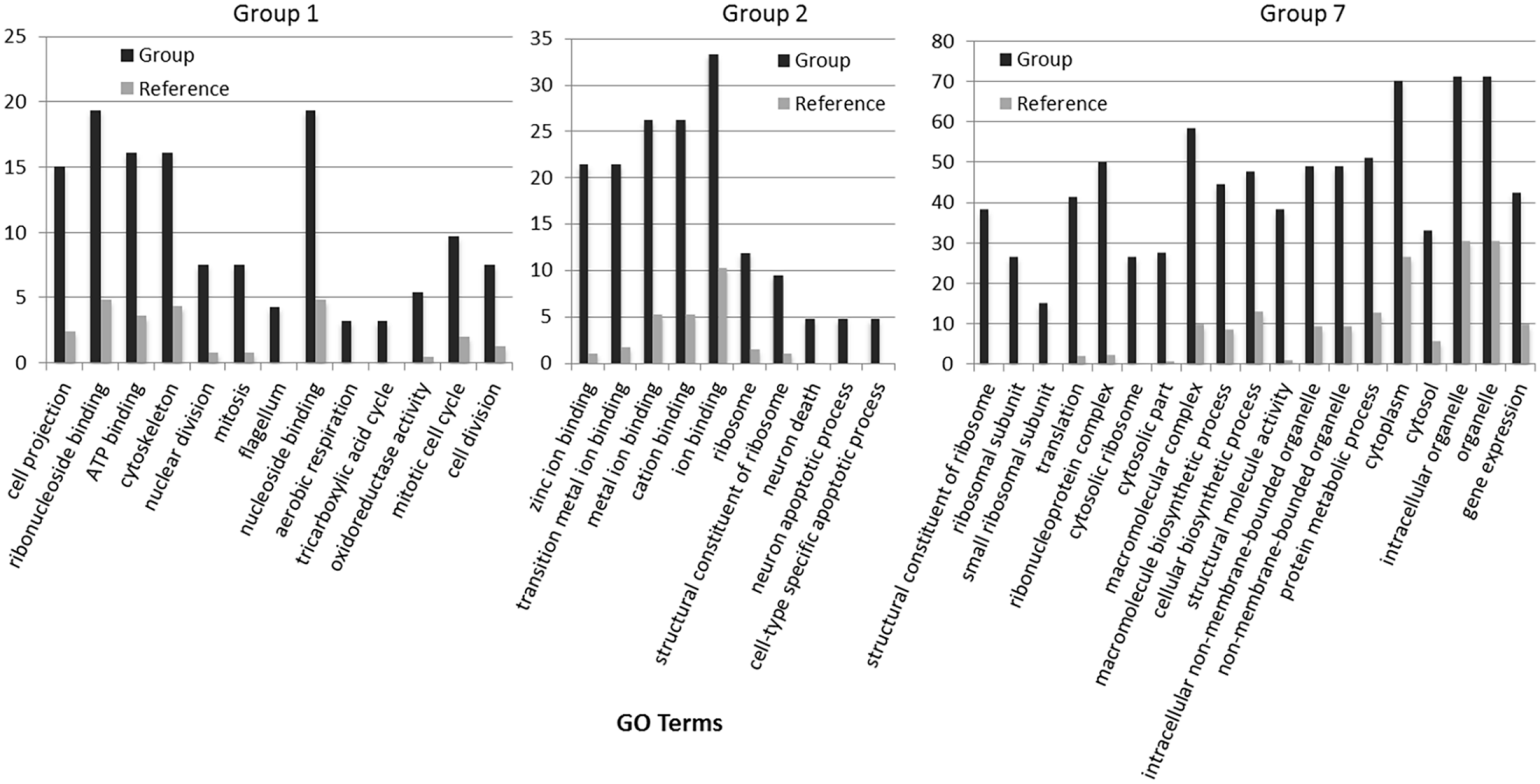
Gene expression heatmap group 1, 2 and 7 GO term overrepresentation. Black bars represent the percentage of genes with the associated GO term (X-axis) within groups 1, 2 and 7. Gray bars represent the percentage of all annotated genes in the *C*. *tobin* genome that have the corresponding GO term. For complete list of overrepresented GO terms in each group, see S2 Dataset.

To better understand how light-entrained gene expression controls cell physiology, the entire transcriptome of *C*. *tobin* was profiled over the light-dark cycle. RNA was collected at 7 time points during the 24 hour cycle (NCBI: SRX1009273) and gene expression levels were calculated (S1 Dataset: 7 time point expression levels for all genes). Genes with the largest expression changes and large transcript variance between two or more time points were identified and binned into 7 groups for analysis (Fig 3 and S2 Dataset complete list of group members). *Group 1* represents genes whose expression peaks midway through the dark period (D6). In this group, transcripts which encode for proteins associated with cell growth and post cell division processes including cytoskeletal changes, and cell projection (microtubular and flagellar components) are overrepresented. *Group 2* displays genes that are highly expressed at the end of the dark cycle (time point D10); overrepresented gene categories include genes encoding proteins responsible for metal ion binding, as well as a variety of mitochondrial genes that encode both transporters and ion exchange machinery (e.g., ferredoxin and rubredoxin). Transcription during this time period also seems to favor the onset of energy molecule and carbohydrate synthesis (Fig 4). Nitrite and phosphate transporters are also observed in this expression cluster, supporting the acquisition of metabolites needed for carbohydrate and fatty acid production. *Group 3* genes are highly expressed during D6 and L1. These genes include the following associated gene ontology (GO) terms: ribonucleoside binding (i.e., purine ribonucleoside binding), GTPase activity, microtubule-based movement, GTP binding, ATP binding, anchoring to plasma membranes, and tubulin. *Group 4* gene expression is highest at the beginning of the light period. Thylakoid, chloroplast, photosynthetic, metabolic, glycolytic, and fatty acid biosynthetic processes are prominent in the GO term list. *Group 5* includes genes that are highly expressed from L4 to D1. Overrepresented GO terms include genes linked to post transcriptional regulation of gene expression, negative regulation of cellular processes, responses to biotic stimuli, and defense responses. *Group 6* genes are upregulated from L1 to L6 and include the GO terms photosystem I and II, chloroplast thylakoid membrane, cell wall, defense response to fungus, response to heat, and ATP binding. *Group 7* has significant over representation of ribosomal subunit gene expression (Fig 4). Upregulation of these genes occurs from the middle to the end of the day, with the majority of members showing maximum expression at hour 10 in the light cycle. Though ribosomal sequences represent < 0.5% of genes annotated, over 38% of genes identified in this group are structural constituents of ribosomes. It is established that ribosomal accumulation usually occurs during the G1 phase of the cell cycle, thereby fostering the production of new biomass ultimately needed to support cell division [32].

Light is a critical factor in the regulation of algal growth [33–35]. As seen above, photoperiod drives a wide range of gene expression rhythms in *C*. *tobin*. The predictability of these temporal programs provides several excellent metabolic targets, such as lipases, for genetic engineering [36,37] and may be of special interest to commercial growers who are dependent on seasonal light availability to serve large-scale algal production efforts.

### Fatty acid biosynthesis

**Fig 5:**
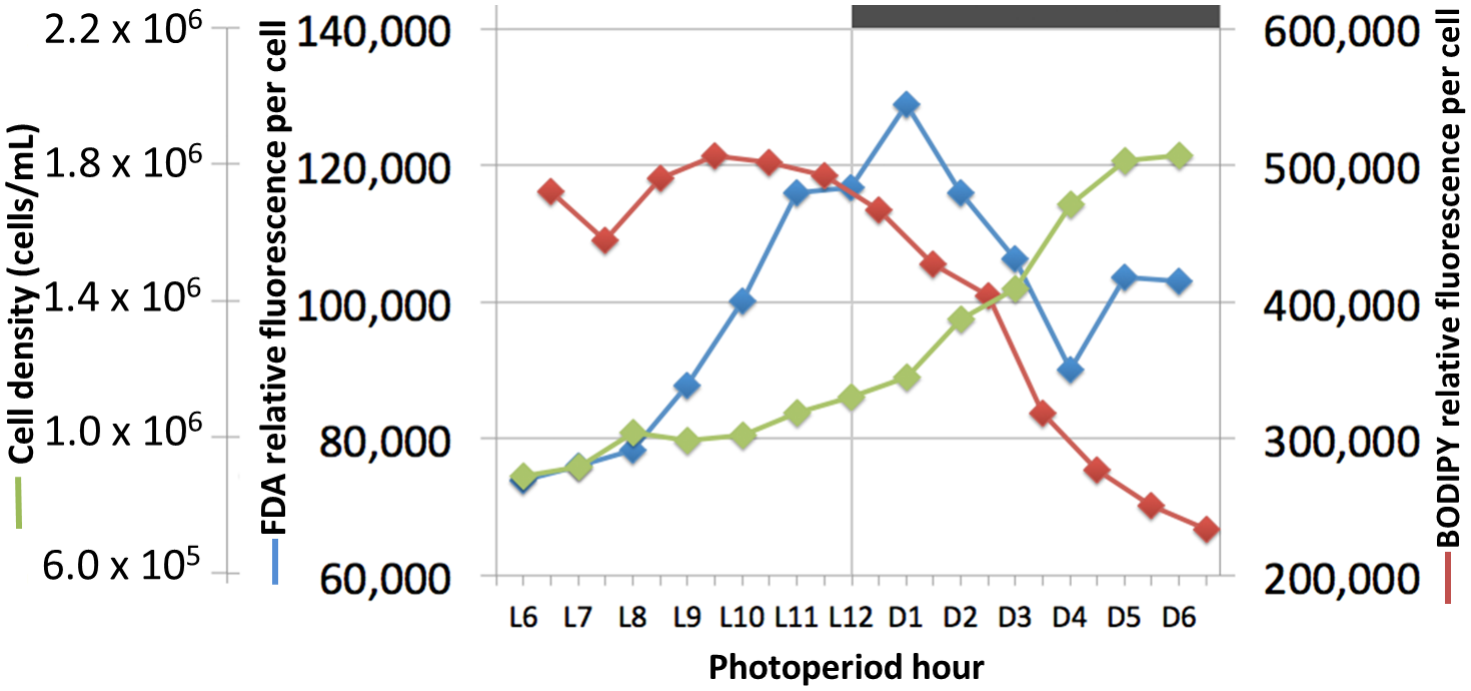
Cell growth, lipid synthesis and lipase activity in a *Chrysochromulina tobin* culture maintained on a 12 hr light: 12 hr dark photoperiod. Cell count (green; n = 3); neutral lipid quantity measured by BODIPY 505/515 dye incorporation (red; n = 2); lipase/esterase activity measured using fluorescein diacetate (blue; n = 3).

The high fatty acid content of algae such as *C*. *tobin* can be harnessed as a source for valuable products such as nutraceuticals, alternative energy, or plastics [37,38]. Additionally, the abundant and complex fatty acid composition of haptophytes has historically made these algae highly valued for aquaculture feedstocks and contributed to their broad currency as a food source in aquatic ecosystems [39]. *C*. *tobin* produces almost half of its dry weight as lipids [22], much of which is stored in the prominent lipid bodies of each cell. Because lipid body biogenesis is simple (only two lipid bodies) and predictable (regulated by the cell cycle), *C*. *tobin* is a viable model organism to study the metabolic processes associated with the genesis of this organelle. Indeed, flow cytometric analysis of *C*. *tobin* cells stained with the lipid dye BODIPY 505/515 shows fatty acid content per cell increases during the light and decreases during the dark phases of cell growth (Fig 5). To gain insight into the potential relationship between the observed changes in lipid body size and fatty acid biosynthesis, the regulation of genes important to fatty acid production and loss were inventoried as cells progressed through a single light/dark photoperiod.

**Fig 6:**
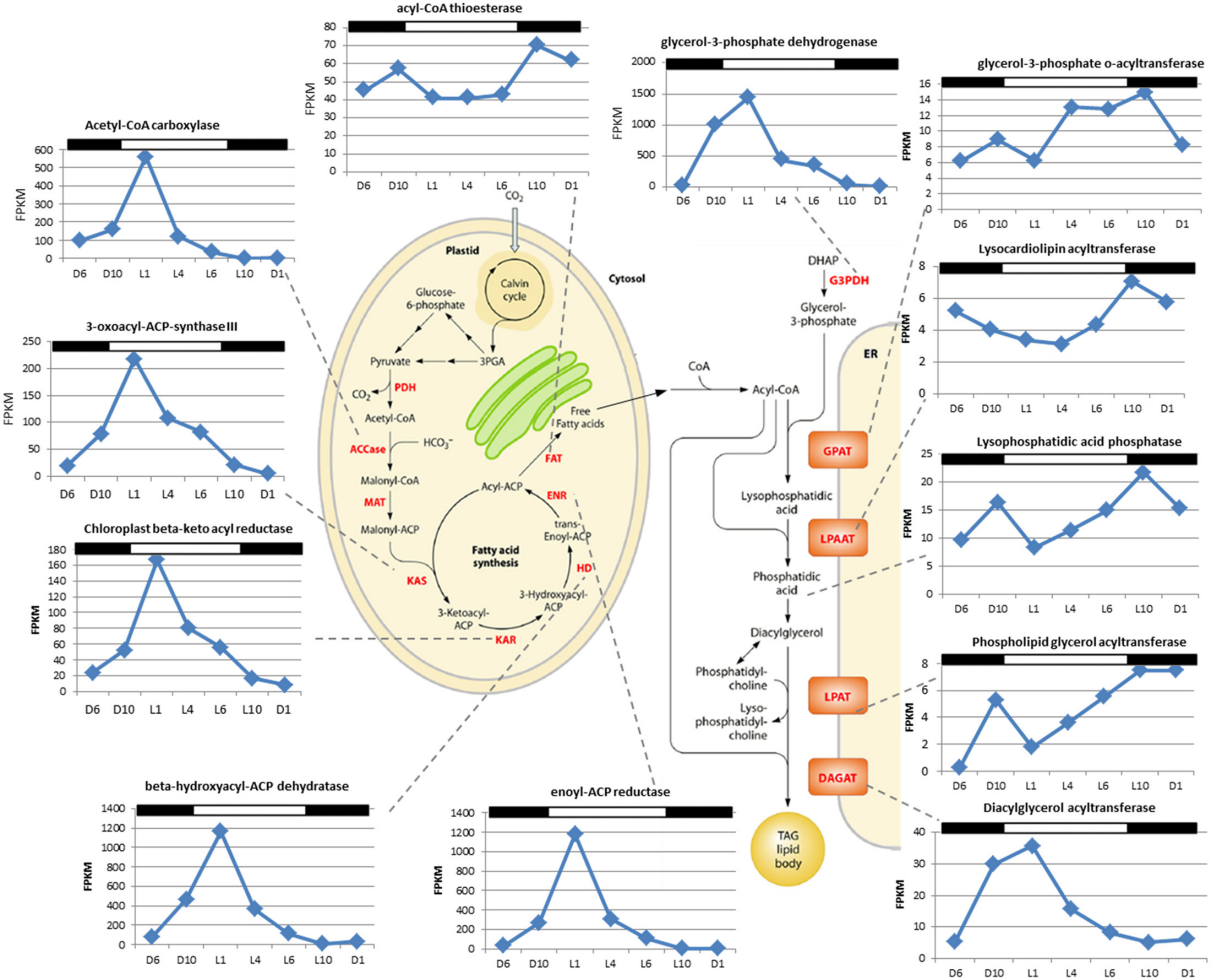
Identification of fatty acid synthesis genes of *Chrysochromulina tobin* and corresponding RNA transcript FPKM over a 12 hr light: 12 hr dark photoperiod. The genes identified as fatty acid synthesis genes and the corresponding chloroplast and endoplasmic reticulum pathway schematic (from [37]).

*C*. *tobin* fatty acid biosynthesis pathways are presented in Fig 6 (after [37]). Two major gene expression patterns are notable. The first pattern relates to genes associated with chloroplast-localized fatty acid synthesis, while the second involves transcripts encoding enzymes that convert fatty acids into triacylglycerols (TAGs) after chloroplast export (Fig 6). Transcripts that encode enzymes for converting Acetyl-CoA to Acyl-ACP (e.g., ACCase, acetyl-CoA carboxylase [Ctob_003321]; KAS, 3-oxoacyl-ACP synthase III [Ctob_004088]; KAR, beta-ketoacyl-ACP reductase [Ctob_008890]; HD, beta-hydroxyacyl-ACP dehydratase [Ctob_006212]; ENR, enoyl-ACP reductase [Ctob_006649]) have increased transcript abundance at the end of the dark photoperiod. This expression pattern also applies to the glycerol-3-phosphate dehydrogenase [Ctob_011737], the enzyme required for readying the glycerol in triacylglycerol (TAG) products. Expression initiates at D10 and peaks at L1 with FPKM (fragments per kilobase of exon per million fragments mapped) [40] peaking at 3 to 500 fold over the lowest value for the aforementioned genes. This expression variability suggests that production of triacylglycerol (TAG) related gene transcripts may be minimal during the dark period.

In the second pattern observed, a gradual increase of transcripts encoding enzymes that convert acyl-CoA to TAG products is observed from L4 to L10 (e.g., GPAT, glycerol 3-phosphate-o-acyltransferase [Ctob_005527]; LPAAT, lysocardiolipin acyltransferase [Ctob_003757]; lysophosphatidic acid phosphatase [Ctob_007879]; LPAT, phospholipid glycerol acyltransferase family protein [Ctob_012317]; DAGAT, diacylglycerol acyltransferase family protein [Ctob_008970]). FPKM value fold change is more modest for these ER-associated transcripts, ranging from 2–4 fold increased transcript abundance during the peak at L10. Interestingly, the last step (DAGAT) of this ER associated metabolic pathway appears to follow the chloroplast gene expression pattern in that transcript expression peaks at L1.

Besides differing in the timing of peak transcript abundance and fold-increase, there is a significant difference between the overall transcript levels for the two groups described above. Chloroplast associated processes are generated by very highly transcribed genes with maximum FPKMs ranging from 70 to almost 1500, compared to the ER related processes that have maximum FPKM transcript levels about 8 to 35. These processes may contribute to the rate limiting steps of TAG production, and suggest that ER related reactions may be candidates for overexpression experiments.

Fatty acid elongase and desaturase transcripts (S2 Fig) appear to follow the trend of the chloroplast associated gene products, in that genes encoding these enzymes consistently peak in their expression at the L1 time point. However there is great variance among overall transcript levels for each gene queried.

In contrast, the gene expression patterns of lipases and esterases parallel those seen in the ER related lipid biosynthesis genes—slowly increasing in the light portion of the photoperiod (S3 and S4 Figs). Similarly, when fluorescein diacetate is used to monitor lipase/esterase levels in synchronized *C*. *tobin* cultures, a slow increase in enzymatic activity is seen from L6 to the onset of dark, followed by a rapid decline (Fig 5). This change in enzyme activity correlates with the concentration of lipid found in the cell. Incorporation of the fluorescent dye BODIPY 505/515 shows lipid levels to peak at ∼L9, and then quickly drop as cells enter the dark phase of growth. Taken together, shifts in gene expression, enzymatic activity and lipid concentration suggest that a sequential cascade of metabolic programs (fatty acid synthesis up-regulation in the light, and lipase activity in the dark) drive significant and predictable changes in *C*. *tobin* fatty acid storage levels, and that these changes are reflected in the lipid body volume noted above (Fig 2).

**Fig 7:**
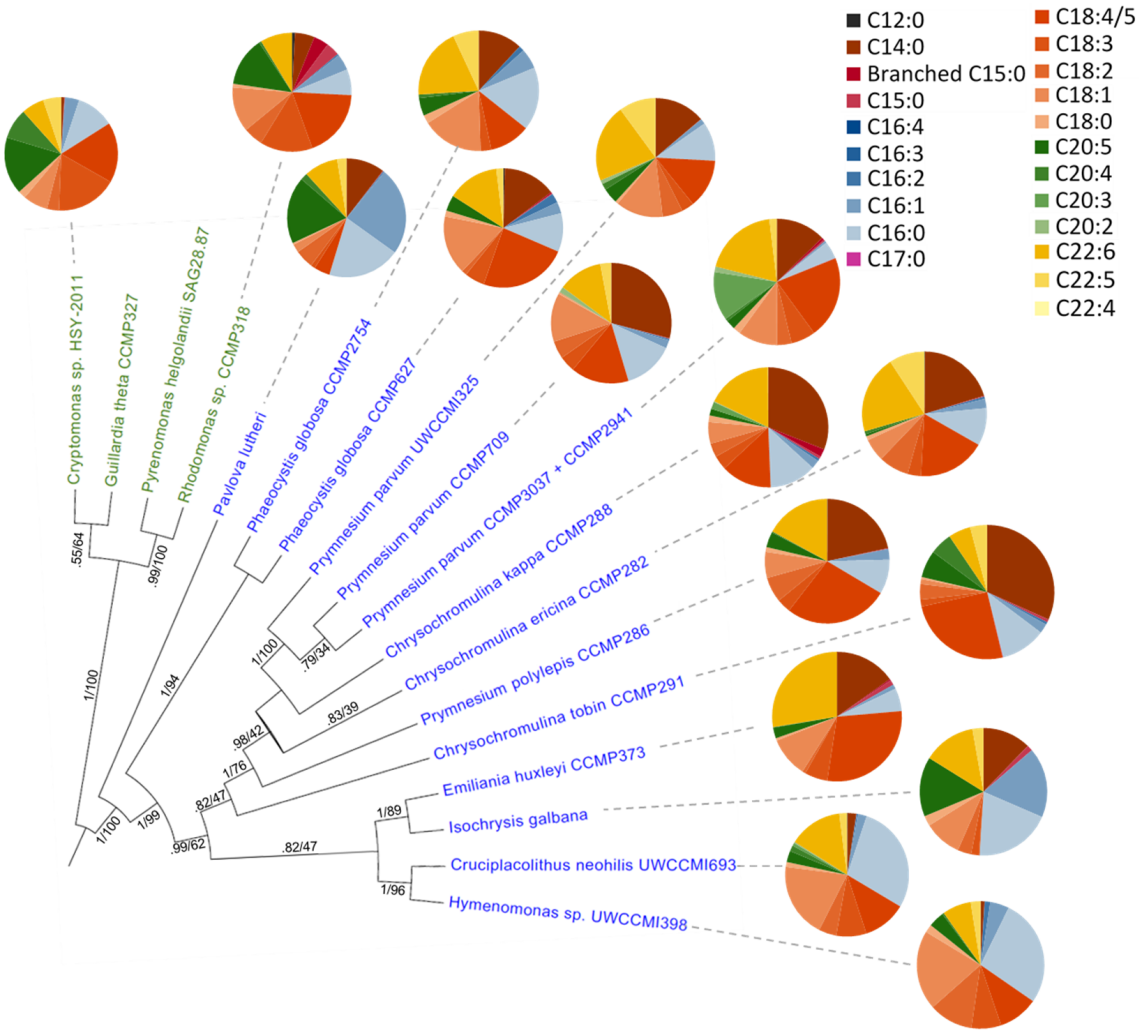
Fatty acid content across haptophyte (blue text) and cryptophyte (green text) algal species. Data collected using GC/MS analysis [41] of total fatty acids. Circle graph wedges represent the proportion of individual fatty acid types. The Bayesian species tree is inferred from an 857 bp alignment of *psb*A nucleotide sequence (S4 Dataset), with posterior probability and maximum likelihood bootstrap support shown for each node.

The data presented here give insight to *C*. *tobin* fatty acid biosynthesis. However, as seen in Fig 7, not all haptophytes generate the same quantity or type of fatty acids, even when organisms are maintained under similar physiological conditions. Gas chromatography-mass spectrometry (GC/MS) data presented in Fig 7 and S3 Dataset, comparing fatty acid profiles obtained from a broad sampling of haptophytes clearly demonstrate that even those haptophytes clustered within a specific taxonomic rank may greatly differ in the type and amount of lipids present. Given that biological findings often lead to metabolic engineering efforts, such information potentially facilitates engineering approaches in commercial algal applications.

### Defense systems

*C*. *tobin* has a war chest of defense related genes. The requirement for defense systems is two-fold: first, the alga may need to cope with potentially harmful eco-cohorts, and second, because *C*. *tobin* is mixotrophic and actively phagocytotic, ingested prey must be neutralized. A variety of these putative defense mechanisms identified in *C*. *tobin* are described below.

#### Polyketides

Polyketides are synthesized from acetyl- or malonyl-CoA, which are also substrates of fatty acid biosynthesis. Polyketide synthase (PKS) pathways that occur in both prokaryotic and eukaryotic organisms generate a variety of biologically active compounds ranging from antibiotics to toxins. Given that polyketide-related toxins are sometimes associated with haptophyte harmful algal blooms [42], we queried the *C*. *tobin* genome for the presence of PKS genes.

**Fig 8:**
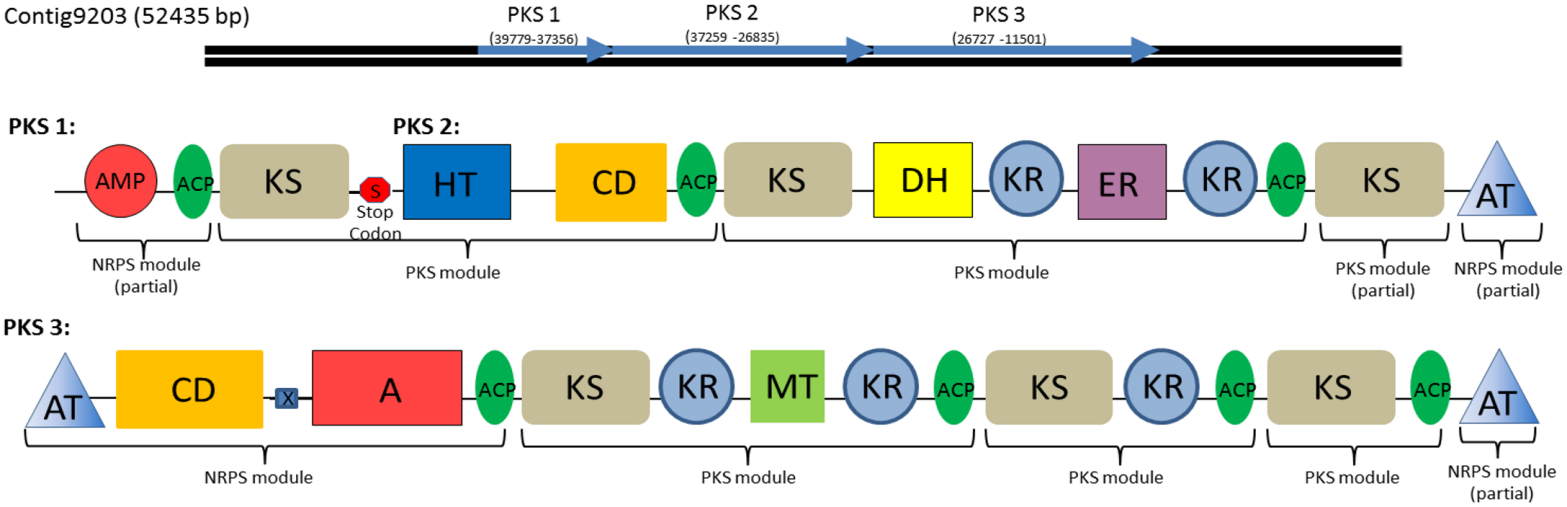
Type I Polyketide synthase and PKS-NRPS hybrid domains found in the *C*. *tobin* genome. These gene clusters each represent one of three polyketide synthesis domains found on a single assembled contig of over 50,000 bp in length. Domains 1 and 2 are separated by a single stop codon whereas domain 3 is separated by a stop codon and is frame shifted. Brackets indicate individual PKS and NRPS modules. Modules contain the following components: adenylation domain (A); AMP binding domain (AMP); Acyl carrier proteins (ACP); acyltransferase (AT); condensation domain (CD); dehydratase (DH); enoyl reductase (ER); crotonase/Enoyl-Coenzyme A (CoA) hydratase family (HT); ketosynthase (KS) methyltransferase (MT); stop codon (S); HxxPF repeat domain (X).

Similar to findings in the previously sequenced *E*. *huxleyi*, *C*. *tobin* encodes polyketide synthase genes. In general, Type I PKSs are large genes (5–25 kb) comprised of multiple “modules” (Fig 8) [43,44]. Each module contains multiple protein active sites, each of which performs a polyketide chain modification. Type I modular PKS minimally contain a ketosynthase catalytic domain (KS), an acyl transferase domain (AT), and an acyl carrier domain (ACP), though the domains found in *C*. *tobin* PKSs are more complex.

In addition to polyketides, many organisms including algae also use nonribosomal peptide synthetases (NRPS) to produce non-ribosomal peptide products. These peptide products are synthesized independently of ribosome protein production pathways and comprise a variety of biological compounds such as antibiotics or toxic compounds [45,46]. NRPS genes, like PKS genes, produce large proteins with multi-functional domain structures. While a standalone NRPS pathway was not found in *C*. *tobin*, we did observe the presence of NRPS modules associated with PKS domains, suggesting the presence of a PKS/NRPS hybrid system [47], allowing for potentially novel bioproduct production.

The identified NRPS modules were found within large open reading frames of a potential PKS gene (Fig 8). A total of three ORFs are observed to occur contiguously. Within PKS 3 (Fig 8), a polypeptide adenylation NRPS domain is located adjacent to a PKS domain. Additional NRPS domains are found upstream of the adenylation domain, including an NRPS specific condensation domain and HxxPF repeat domain. Both termini of PKS 3 are acyl transferase (AT) domains that facilitate the transfer of an amine to growing acyl chains [48]. A similar AT chain is found at the C terminus of PKS 2 as well, supporting speculation that all three domains may interact to create a single product. Smaller, single function Type III PKSs or remnants of Type I PKS domains are also observed (S3 Table).

To our knowledge, this is the first time a hybrid PKS-NRPS has been described in an algal species. Hybrid PKS-NRPS pathways have been identified previously in bacteria, cyanobacteria and fungi [49]. Such observations are significant, given the extensive interest in identifying new therapeutic compounds that are produced by either PKS or NRPS hybrid pathways. For example, fungal products produced by PKS-NRPS hybrid pathways include Fusarin C, a toxin which has been shown to be an estrogen agonist [50] and carcinogen [51], as well as Pseurotin A [52], a chitin synthase inhibitor. Bacterial products from hybrid pathways include broad-spectrum antibiotics [53]. Additional investigation of published as well as in-progress algal genomes is warranted to identify these potentially useful gene complexes.

#### Macrolides

Tylosin is a member of the polyketide-derived macrolide family of antibiotics whose activity (inhibition of peptidyl transferase) is derived from a large macrocyclic lactone ring to which one or more deoxy sugars are attached [54]. Tylosin or a tylosin-like antibiotic is likely produced by *C*. *tobin*, given the presence of multiple genes (Ctob_012974, Ctob_004215, and Ctob_007333) with homology to macrocin-O-methyltransferase (EC 2.1.1.101), the enzyme responsible for the final step of tylosin synthesis. Macrocin-O-methyltransferase genes are also conserved in the *E*. *huxleyi* genome sequence, but described only as hypothetical proteins. The complete tylosin biosynthesis pathway has only been characterized in the Actinobacteria, *Streptomyces fradiae* [55–57].

A second example of a macrolide antibiotic is erythromycin, which also inhibits protein synthesis by blocking peptide chain elongation [58]. Many bacteria are able to reduce their susceptibility to this antibiotic by neutralizing the molecule. This task is accomplished by erythromycin esterase, an enzyme that catalyzes the hydrolysis of the macro-lactone ring in the antibiotic. *C*. *tobin* encodes an expressed gene (Ctob_004084) whose protein product (523 amino acids) has high identity to well-studied bacterial erythromycin esterase enzymes. Functionally important amino acids of erythromycin esterase are completely conserved in identity, including H46/56 (*Massillia* sp. JS1662 numbering/*C*. *tobin*) that is indispensable for catalytic function, and the 9 amino acids that are critical for maintaining the active site pocket of the enzyme (S5 Fig) [58]. Interestingly, to date, this gene has been identified in some cyanobacteria (*Fischerella muscicola; Fischerella* sp. PCC9431; *Cylindrospermum stagnale*) but no other eukaryotic algal species.

#### Antimicrobial peptides

Peptide antibiotics can be synthesized as a defense against bacterial attack. *C*. *tobin* encodes three putative antimicrobial gene products that are all located on contig 8288. A 40 amino acid encoding-repeat is found in each open reading frame. Ctob_015847, Ctob_015848 and Ctob_015849 have 9, 3, and 5 copies of the 40 residue repeat respectively. When compared to Ctob_015847, the Ctob_015848 and Ctob_015849 genes have BLASTP e-values of 4e-69 and 2e-101 respectively. All genes are expressed in an identical pattern and are equally abundant over the 12 hour light: 12 hour dark photoperiod. When the *C*. *tobin* genes are queried against the NCBI non-redundant protein database, the highest identity match is an antimicrobial peptide found in the haemolymph of the insects *Riptortus pedestris* and *R*. *clavatus*. The peptide found in *C*. *tobin* is similar in size to that found in these hemipterans (e.g. 47 amino acids) and is rich in proline, as found in the peptides of *Riptortus*, honeybees, and in fruit flies [59]. The broad interest in alternative antimicrobials has prompted a significant bioinformatics effort to accurately identify these compounds and ultimately to predict their antibacterial targets. Using the curated Collection of Anti-Microbial Peptides (CAMP) [60] antimicrobial peptide prediction tool (http://www.camp.bicnirrh.res.in/index.php), all three *C*. *tobin* sequences scored between 0.97 and 1.0 probability (1.0 being the highest score), suggesting a very high likelihood of having antimicrobial activity [61]. Although antimicrobial activity has been reported to be present in the extracts of several algal species [62,63], to our knowledge, no one has identified such potentially antibacterial peptide sequences in algae. Pragmatically, these novel antimicrobial peptides could be used in large-scale algal growth systems, providing an inexpensive method for moderating bacterial contamination.

#### Multidrug and toxic compound extrusion proteins

Multidrug and toxic compound extrusion proteins (MATEs) are large, multi-pass membrane peptides that are found in both prokaryotes and eukaryotes [64,65]. MATE proteins appear to have a multiplicity of functions—they have been shown to mediate multi-drug resistance, assist in the removal of metabolic waste products, and affect the extrusion of xenobiotics from cells [66,67]. These proteins function by generating a Na+/H+ electrochemical gradient that impacts metabolite efflux. To date, five *C*. *tobin* MATE genes have been identified (Table 2). These genes do not appear to be recent duplication products since each gene has only low sequence identity to one another. All genes are transcribed, with transcript abundance ranging from high to low (i.e., Ctob_006254> Ctob_005630> Ctob_012228> Ctob_002830> Ctob_015454), and each having a specific expression pattern in response to the 12 hr light: 12 hr dark photoperiod on which the *C*. *tobin* cultures were maintained (S1 Dataset). Homologues to genes encoding MATE proteins have not been commonly identified in eukaryotic algae. Their functional contribution to algae remains unknown. Interestingly, a DinF-like MATE domain is found in two of the *C*. *tobin* proteins. It has been suggested that MATEs having this motif might serve to protect cells against oxidative stress [68].

**Table 2:** *Chrysochromulina* MATE domain detail.

MATE gene	Domain Hits	E-value*
MATE efflux family protein (Ctob_005630)	MATE-DinF-like	5e^-47^
	Na+-driven multidrug efflux pump-Defense mechanisms	2e^-38^
MATE efflux family protein (Ctob_006254)	MATE-like-1	3e^-55^
	Na+-driven multidrug efflux pump-Defense mechanisms	1e^-35^
Multi antimicrobial extrusion family protein (Ctob_012228)	MATE-Eukaryotic	8e^-101^
	Na+-driven multidrug efflux pump-Defense mechanisms	1e^-35^
MATE efflux family protein(Ctob_002830)	MATE-DinF-like	5e^-49^
	Na+-driven multidrug efflux pump-Defense mechanisms	5e^-26^
Multidrug and toxin extrusion protein 2 isoform 3(Ctob_015454)	MATE-Eukaryotic	2e^-53^
	Na+-driven multidrug efflux pump-Defense mechanisms	6e^-34^

*Based of the NCBI Conserved Domains database from BLASTP ‘nr’ database query

### Alternative energy capture and nutrient sourcing

Many algae have evolved methods that augment survival either by optimizing energy capture [69] or by circumventing the need for synthesizing required compounds [70]. Both of these options appear to be used by *C*. *tobin*.

#### Non-photosynthetic light capture

Microbial Type 1 rhodopsins are a class of proteins that enable the non-photosynthetic transduction of solar energy for use in biological processes [71]. These light-activated, membrane-integral proteins are comprised of seven trans-membrane alpha helices that surround an all-trans retinal chromophore. Rhodopsins perform an array of cellular functions via evolutionary modification of their protein structures; serving as proton or chloride pumps, cation channels or photosensors. Though once designated as “microbial rhodopsins”, these proteins are found in both prokaryotes and eukaryotes [72].

We document a xanthorhodopsin in *C*. *tobin* (Ctob_004469) which is one of the most recently identified members of the rhodopsin family. Similar to other rhodopsins, xanthorhodopsins covalently link to retinal as a protonated Schiff base via a lysine in transmembrane seven [69,73]. Unlike other rhodopsins, xanthorhodopsins also non-covalently associate with a carotenoid, forming a dual chromophore system [74] that augments rhodopsin function. For example, energy transfer by the carotenoid antennae of *Salinibacter rubrum* is ∼40% [75]. The light energy harnessed by xanthorhodopsin powers a proton pump [76].

**Fig 9:**
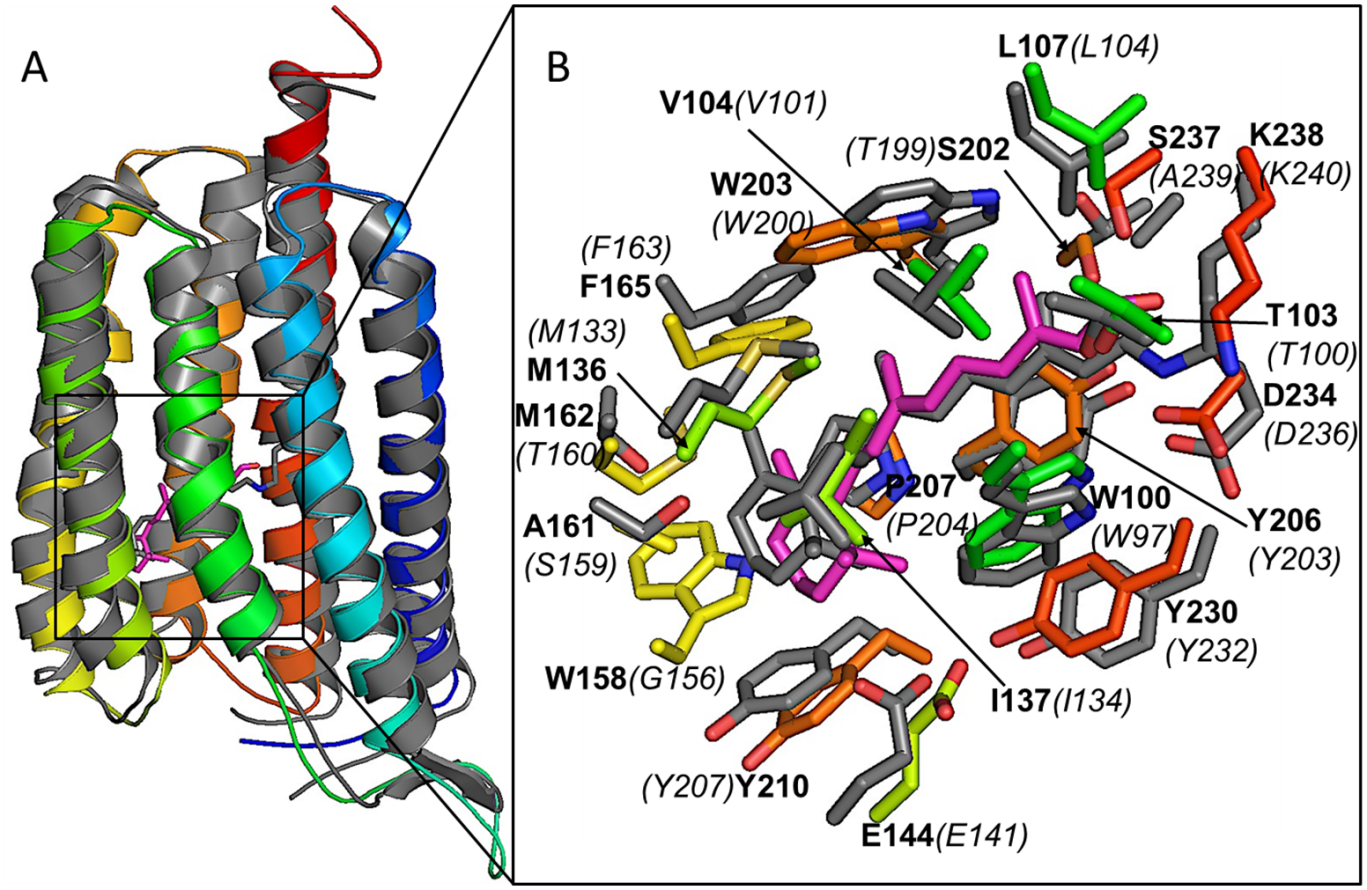
Xanthorhodopsin structural modeling. (A) Comparative model of *C*. *tobin* xanthorhodopsin (in color, blue as N-terminus and red as C-terminus) overlayed on the structural template (PDB code 3DDL, grey). A docked retinal molecule (magenta) and conjugated retinal (grey) in structural template are also shown. (B) Enlarged view of the ligand pocket in the aligned structures. Docked retinal molecule (magenta) and its first shell amino acids (in colors) are shown along with conjugated retinal (grey) and its first shell amino acids (in grey) confirm existence of a retinal compatible pocket in *C*. *tobin* xanthorhodopsin. Labels are based on *C*. *tobin* xanthorhodopsin sequence (bold characters) and 3DDL structural template (italics and in parentheses).

Sequence identity and three-dimensional similarity in molecular architecture between *C*. *tobin* and *S*. *rubrum* xanthorhodopsin 3DDL crystallographic structure (1.9 A resolution [75]) provide strong evidence that the two proteins are related (Fig 9). *C*. *tobin* retains critical residues (3DDL /*C*. *tobin* numbering) including Asp 96/99 (proton acceptor), Leu 104/107 (spectral tuning; green), Glu 107/110 (proton donor), and Lys 240/238 (retinal binding). Residue variations around the trimethylcyclohexene group of retinal (Trp158 and Met162) in *C*. *tobin* were aligned with much smaller side chains (Gly162 and Thr160) in the template (3DDL), resulting in a relatively tighter pocket for retinal in *C*. *tobin* xanthorhodopsin.

The single *C*. *tobin* xanthorhodopsin gene is highly and temporally expressed when monitored over the 12 hour light: 12 hour dark photoperiod (S6 Fig and S1 Dataset). Transcript abundance increases significantly from D6 to D10, then falls precipitously as the light period ensues.

Several genes supporting rhodopsin function are encoded in the *C*. *tobin* genome. Most notable is the occurrence of two bacterio-opsin activator genes (*bat*) (Ctob_004970 and Ctob_007302). Studies in bacteria [77] suggest that when cells experience low oxygen tension, *bat* gene transcription is induced, and that the resultant protein product influences the up-regulation of rhodopsin gene expression. Transcription of both *C*. *tobin bat* genes (both contain PAS domains) is low in the dark phase of growth, slowly increases at the onset of light, reaches a maximum at L6, and remains elevated until onset of the dark period (S7 Fig and S1 Dataset). Additional contribution of genes ancillary to rhodopsin function include those that produce enzymes important to retinal synthesis such as lycopene beta cyclase (Ctob_003991), 15’15’ beta-carotene dioxygenase (Ctob_007450) and retinol dehydrogenase (Ctob_005465).

**Fig 10:**
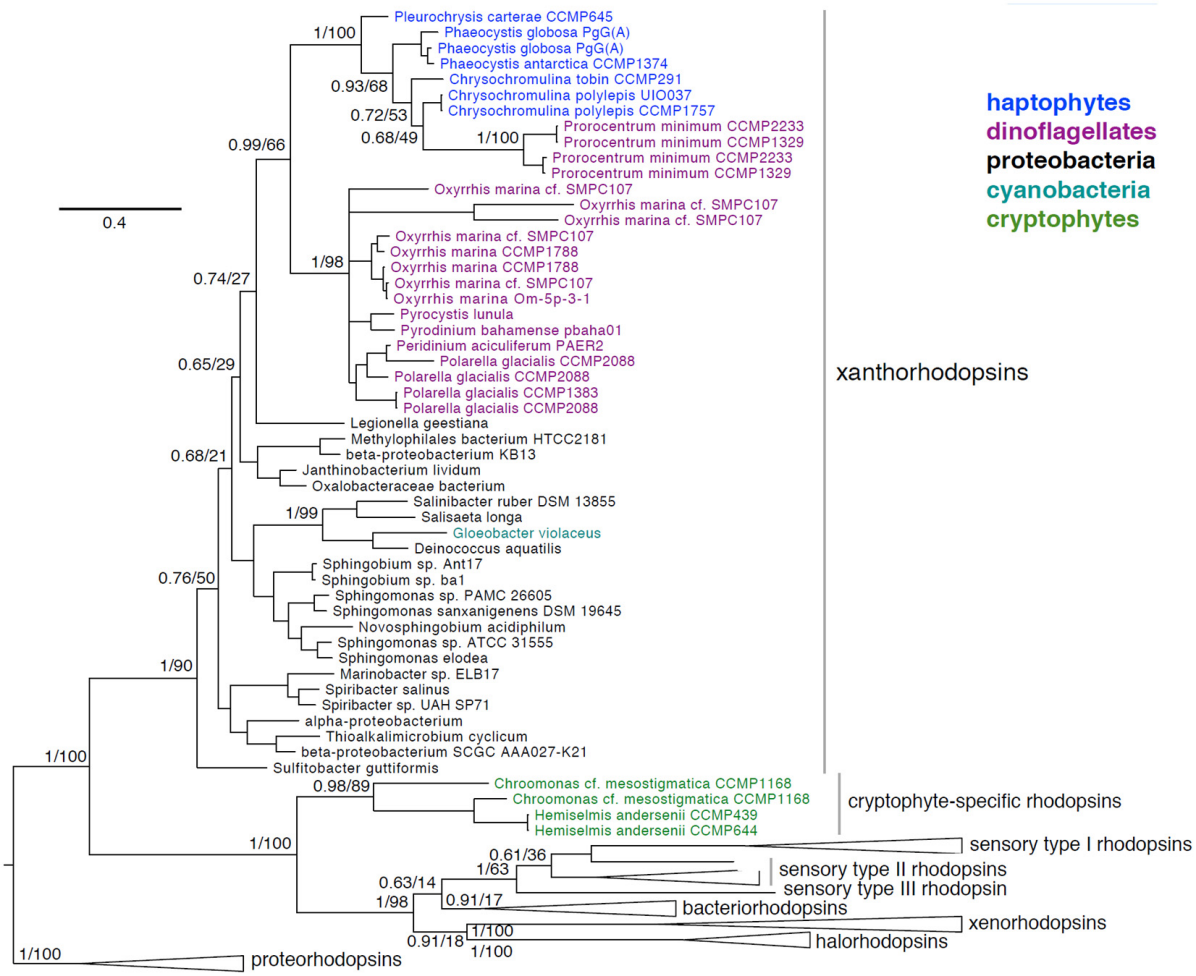
Phylogenetic placement of haptophyte, dinoflagellate, and cryptophyte xanthorhodopsins. Bayesian phylogeny inferred from a 231 amino acid alignment of rhodopsins, with posterior probabilities and maximum-likelihood bootstrap support values shown at key nodes. Clades of xanthorhodopsins, novel cryptophyte-specific rhodopsins, sensory type I, II, and III rhodopsins, bacteriorhodopsins, xenorhodopsins, halorhodopsins and the proteorhodopsin outgroup are indicated.

The identification of algal rhodopsin variants represents a new platform for discovery. Previously, xanthorhodopsins were shown to occur solely in several dinoflagellate species [69,78]. We confirm and extend this observation (S5 Dataset and Fig 10) using publicly available genomes and transcriptomes (Marine Microbial Eukaryote Transcriptome Sequencing Project) [3]. In addition to incorporating new and previously identified xanthorhodopsins from dinoflagellates, we document xanthorhodopsins from several taxonomically diverse haptophytes including *Chrysochromulina tobin* (Prymnesiales B2 clade), *Prymnesium polylepis* (Prymnesiales B1 clade), *Phaeocystis antarctica* (Phaeocystales), *Phaeocystis globosa* (Phaeocystales), and *Pleurochrysis carterae* (Coccolithales). Also documented are rhodopsins in the cryptophytes *Chroomonas mesostigmata* (Pyrenomonadales) and *Hemiselmis andersenii* (Cryptomomadales) that do not cluster with xanthorhodopsins, and may represent new, yet undescribed rhodopsin variants.

As seen in Fig 10, eukaryotic xanthorhodopsins form a well-supported clade (0.99/66) whose exact placement within proteobacterial xanthorhodopsins is uncertain (0.74/27). Within eukaryotic xanthorhodopsins, there appears to be strongly supported sister groups of dinoflagellate xanthorhodopsins and haptophyte xanthorhodopsins. The sister relationship of haptophyte and dinoflagellate xanthorhodopsins may reflect their acquisition via lateral gene transfer (LGT) from similar proteobacterial species or, alternatively transfer between the two groups. The dinoflagellate *Prorocentrum minimum* appears to have obtained a *C*. *tobin*-like xanthorhodopsin in a unique LGT event. Dinoflagellates are known to have acquired many genes by LGT [79], although alternative explanations for these data must be considered such as incomplete taxon sampling or homoplasy due to the high sequence divergence rates noted among dinoflagellates. We postulate that xanthorhodopsins were acquired early in the evolution of haptophytes and dinoflagellates (including the ancestral dinoflagellate, *Oxyrrhis*), given the presence of this protein in diverse lineages of these groups.

#### Carbohydrate synthesis

Our studies first demonstrated that “red” (red algae and algae that obtained their chloroplasts from a rhodophyte via serial endosymbiosis) and “green” (terrestrial plants and chlorophytic algae) Ribulose-1,5-bisphosphate carboxylase (RuBisCOs) differed in coding location and function [80–82]. Given that “red” RuBisCO had a proteobacterial identity, we propose that this *rbcL*-*rbcS* chloroplast-encoded gene set was a product of lateral gene transfer. For optimal catalytic activity RuBisCO requires the companion enzyme RuBisCO activase [83]. Recently the RuBisCO activase of the proteobacterium *Rhodobacter sphaeroides* was shown to activate “red” RuBisCO [84,85]. The *cbbX* gene is usually, located downstream from the *rbcL*-*rbcS* cluster in the red algal lineage chloroplasts. Given their significant sequence differences “red” and “green” RuBisCO activases most likely represent different evolutionary products.

*C*. *tobin* encodes two *cbbX* (RuBisCO activase) genes; one chloroplast (ChtoCp_00130) and one nuclear (Ctob_015604). The *C*. *tobin* nuclear encoded gene generates a protein 115 (putative signal peptide) and 10 residues longer at the amino and carboxyl termini respectively, than the chloroplast-encoded gene product. Both proteins conserve essential amino acids and motifs found in the *R*. *sphaeroides* “red activase” protein including (*R*. *sphaeroides /C*. *tobin* nuclear CbbX numbering); 35-36/141-2(N-linker); 76-83/183-190 (Walker A motif), 131-142/238-249 (Walker B motifs), 114-116/221-223 (pore loop), 175-179/282-286 (sensor 1 domain), 247-249/354-356 (sensor 2 domains), 194/301 (arginine finger), and 198/305 (the histidine sensor which is “unique to *cbbX* sequences“) [81]. *Chrysochromulina tobin cbbX* nuclear transcripts are abundant and appear to be highly upregulated under the light phase of the light/dark photoperiod. Similar to *C*. *tobin*, copies of *cbbX* were found in earlier studies of the cryptophyte *Guillardia theta* [84], where *cbbX* copies were found in the nucleomorph as well as chloroplast of this alga, followed by observations in the rhodophyte *Cyanidioschyzon merolae* [86] where *cbbX* was seen to occur in the nucleus as well as the chloroplast. Below we expand on preliminary observations that suggested that a broad phylogenetic occurrence of a chloroplast/nuclear *cbb*X coding duality exists in red lineage algae [87].

**Fig 11:**
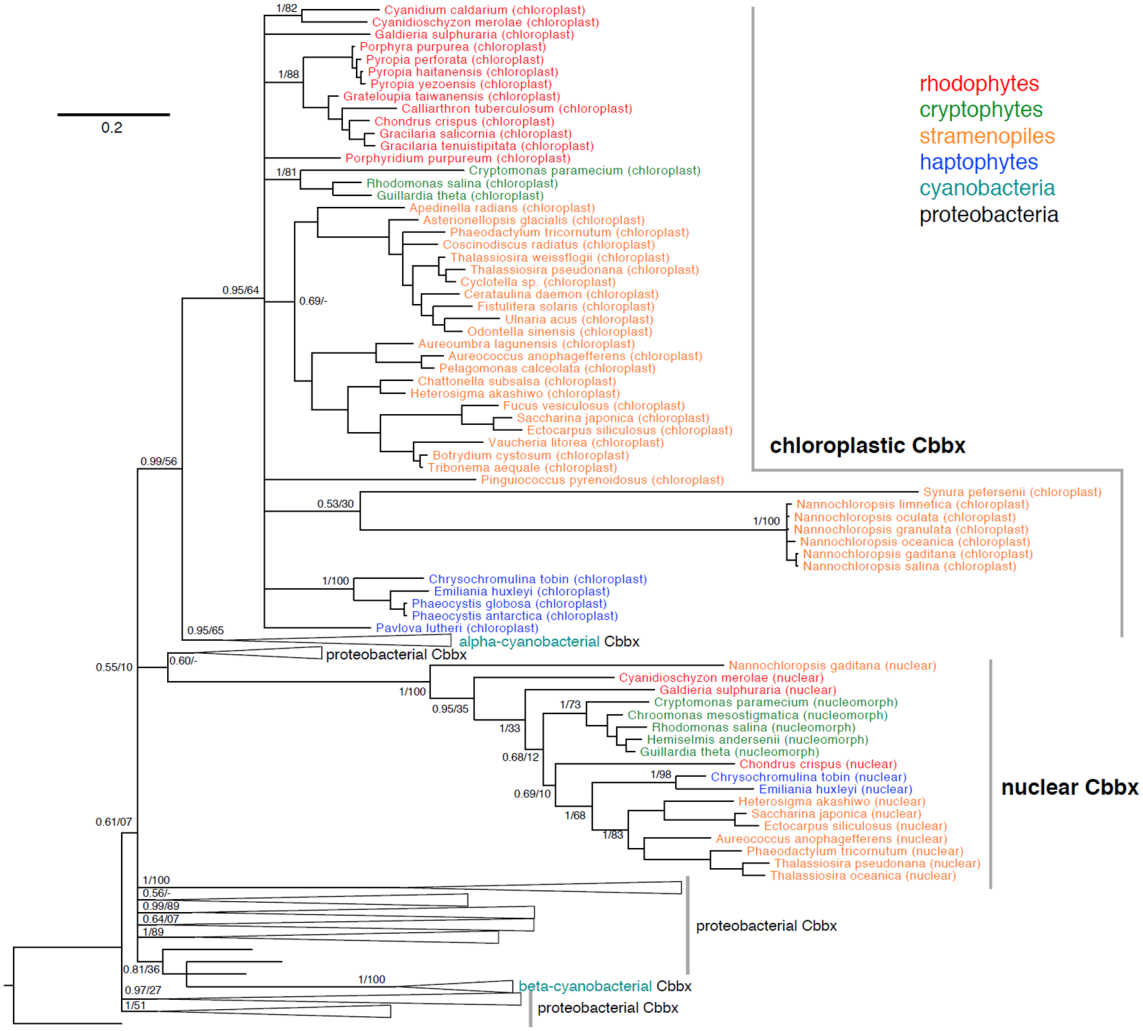
Phylogeny of chloroplast and nuclear CbbX proteins from rhodophytes, cryptophytes, stramenopiles, and haptophytes in a background of cyanobacterial and proteobacterial CbbX proteins. Bayesian phylogeny inferred from 257 amino acids, with posterior probabilities and maximum likelihood bootstrap support shown at key nodes. Alignments in S6 Dataset.

A survey of CbbX sequences shows that both nuclear (or nucleomorph-localized in cryptophytes) and chloroplast CbbX proteins are found in two rhodophytes (*Chondrus crispus*, *Cyanidioschyzon merolae*), two cryptophytes (*Guillardia theta*, *Rhodomonas salina*), two haptophytes (*C*. *tobin*, *Emiliania huxleyi*), and five stramenopiles (*Aureococcus anophagefferens*, *Ectocarpus siliculosus*, *Heterosigma akashiwo*, *Phaeodactylum tricornutum*, *Thalassiosira pseudonana*). Fig 11 also includes chloroplast CbbX proteins for many taxa whose nuclear genomes have not been sequenced. In a phylogenetic context, both the nuclear-encoded and chloroplast CbbX proteins form separate, strongly supported branches, suggesting that *each* originates from a transfer event early in the evolution of red-lineage algae. Earlier researchers attributed the nuclear *cbbX* copy to duplication of the chloroplast gene and subsequent transfer of one gene copy to the nucleus, early in the evolution of the red lineage [84,86]. This hypothesis predicts that chloroplast and nuclear proteins will be sister to one another phylogenetically. However, in our phylogeny incorporating nearly 200 prokaryotic CbbX proteins, the chloroplast CbbXs of red-lineage algae demonstrate a closer association with those of alpha-cyanobacteria than to their nuclear CbbX counterparts, suggesting that the chloroplast and alpha cyanobacterial CbbXs were acquired from a similar proteobacterial lineage. In contrast, the nuclear CbbXs of red-lineage algae are found within proteobacterial genes, albeit with very low support. These data suggest that the nuclear and chloroplast *cbbX* genes of red-lineage algae may have been obtained in separate lateral gene transfer events. We caution, however, that phylogenetic results can be confounded by constraints on the sequence evolution of genes in the chloroplast genome and/or poor phylogenetic signal given the ancient occurrence of such an event.

Why do two highly conserved, nuclear and chloroplast encoded CbbX proteins, persist across wide evolutionary distances in red-lineage algae? The answer may lie in either enzyme structure or function. The *R*. *sphaeroides* activase enzyme is a homohexamer [88]. Whether the algal enzyme is a homo- (nuclear or chloroplast subunits only) or heteropolymer (a mix of nuclear and chloroplast subunits) remains unknown. It is also unclear how enzyme construction/function responds to physiological challenges imposed on the cell. For practical considerations, the presence of two *cbbX* copies within the genomes of commercially important red lineage algae [87] and the dependence of RuBisCO on an associated activase, warrants caution when proposing genetic manipulation of CO_2_ processes in these organisms [89].

#### Auxotrophy

It has been proposed that loss of the ability to synthesize vitamin B_12_ (cyanocobalamin) served as an evolutionary determinant for the emergence of auxotrophy in algae [70]. Though few enzymes require B_12_ as a co-factor, these proteins often serve critical roles in cellular metabolism. Algae that are unable to synthesize B_12_ have two alternatives—either to use enzymes that do not require B_12_ as a co-factor or to import B_12_ from an extracellular source. Indeed, several algae employ the first option. For example, the presence of a B_12_-independent methionine synthase (METE) that catalyzes the regeneration of methionine from homocysteine, has been shown to occur in several algal species (e.g., *Chlamydomonas reinhardtii*, *Micromonas pusilla*, *Chlorella* NC64A) [90]. In contrast, an alternative B_12_-requiring methionine synthase (METH), catalyzing the methionine regeneration reaction, has been identified in algae that are incapable of synthesizing this vitamin [90,91]. Thus these organisms must rely on B_12_ import.

Nutrient-dependence studies, conducted in this laboratory, demonstrate that the addition of B_12_ to *C*. *tobin* culture medium is needed for cell survival. The use of B_12_ by *C*. *tobin* to support metabolic processes is further supported by the genomic presence and expression of several proteins including: methionine synthase reductase (MTRR) (Ctob_012110), an enzyme that regenerates CoI from CoII and is indispensable for METH activity; an adenosyl cobalamin-dependent methymalonyl-CoA mutase (MCM) (Ctob_006813) that catalyzes the isomerization of methylmalonyl-CoA to succinyl-CoA; as well as CBLA (found in transcript assembly only) and CBLB (Ctob_008978) that support the synthesis of adenosylcobalamin—all B_12_ dependent enzymes.

Genome mining shows that this alga solely encodes B_12_-dependent METH (Ctob_004248), but not the B_12_ independent METE enzyme (Table 3). Identification of genes encoding METH and MCM but not METE has been reported to occur in the genomes of several other haptophytes (e.g., *Emiliania huxleyi*, *Prymnesium parvum*, *Chrysochromulina brevifilum*, *Chrysochromulina ericina*, and *Phaeocystis antarctica*) [92]. Whether the METE pathway ever existed in the haptophytes is open to conjecture, given the broad range of METE pathway loss now observed among phylogenetically diverse representatives of this algal assemblage. Interestingly, we have identified the presence of several genes whose products contribute to B_12_ synthesis. *Cob*W (cobalamin biosynthesis protein [Ctob_004248]) occurs in all the haptophytes listed above [92]. Additionally, we find excellent sequence identity of a *C*. *tobin* gene to *cob*S (cobalamin 5’ phosphate synthetase [Ctob_009778]), an enzyme that catalyzes the last step in B_12_ synthesis (also found in *E*. *huxleyi*), as well as *cbi*X (a class II cobaltochelatase [Ctob_001499]), whose product inserts metal into a protoporphyrin ring (also found in *E*. *huxleyi*). Whether the proteins encoded by these genes have been re-purposed or represent evolutionary footprints of a previously functional vitamin B_12_ pathway is not known. Finally, it may be that *C*. *tobin* uses more than one strategy to circumvent the need for B_12_. One method may be the use of alternative enzymes that do not need B_12_ as a co-factor. For example, the ribonucleotide reductases found in this alga are of the Class I type which use a diiron-tyrosyl radical as a metallocofactor, rather than a Class II enzyme that requires the cofactor adensylcobalamin. Alternatively, *C*. *tobin* may obtain B_12_ from an exogenous source. Experiments show increased growth and lipid production occurs when this alga is grown in the presence of its 10-membered bacterial biome (Deodato et al., in prep.). The fact that *C*. *tobin* is phagocytotic adds credibility to the argument that it might be advantageous to acquire a complex co-factor such as B_12_ (needing more than 30 steps for its biosynthesis) from bacterial eco-cohorts, rather than expending the energy to generate such a complex product *de novo*.

**Table 3:** Vitamin B_12_ related genes identified in the *Chrysochromulina tobin* genome.

Gene	Abbreviation	*C tobin genome*
B_12_ independent methionine synthase	METE	Not found
B_12_ dependent methionine synthase	METH	Ctob_004248
Methionine synthase reductase	MMTR	Ctob_012110
Methylmalonyl-CoA mutase	MCM	Ctob_006813
Vitamin B_12_ MT transport (MCM accessory protein)	CBLA	Transcript assembly only
AdoCbl synthesis	CBLB	Ctob_008978

### Chloroplast and mitochondrial genomes

Three recovered contigs represent organellar genome sequence and were removed prior to nuclear gene calling and annotation. Read depth of these organelles suggests a copy number of ∼250 chloroplast and ∼800 mitochondrial copies per haploid cell. Complete *C*. *tobin* mitochondrial and chloroplast genomes are presented in detail elsewhere [30]. Briefly, the 34,288 kb mitochondrial genome encodes 48 genes. This genome has a large 9.3 kb repeat section comprised of three large tandem repeats of ~1.5 kb flanked by smaller repeats—similar to that observed in diatoms [93] and cryptophytes [94]. The 104,518 kb chloroplast genome encodes 145 genes. Similar to rhodophyte, stramenopile and other haptophyte plastids, the *C*. *tobin* chloroplast genome contains a preponderance of small, inverted repeats (rather than tandem repeats that dominate in chlorophytic chloroplast genomes) and several unique genes.

### Conclusions

*Chrysochromulina tobin* represents the second haptophyte whose genomes (nuclear and organellar) have been sequenced. The nuclear genome is small, compact, gene-rich, and provides evidence of lateral gene transfer events that have contributed to the evolutionary restructuring of this taxon. Transcriptomic data over a 24-hour light:dark cycle reveals a photoperiod-linked gene expression program that is linked to key features of metabolism including lipid biosynthesis and degradation. These data provide the basis for using *Chrysochromulina tobin* to study lipid body biogenesis.

Because *C*. *tobin* is phagocytotic, genes encoding host defense were anticipated, and were identified in the form of genes encoding potential antibiotics, antibiotic extrusion proteins, as well as novel antibacterial peptides. *C*. *tobin* also represents the first alga for which a polyketide synthase-non ribosomal peptide synthetase (PKS-NRPS) has been identified. This finding may provide potential routes for the synthesis of useful novel metabolites and therapeutics. *Chrysochromulina tobin* also encodes the first documented xanthorhodopsin in a non-dinoflagellate eukaryote, and led to the identification of equivalent genes in haptophyte, dinoflagellate and cryptophyte species with the cryptophyte rhodopsin-like proteins forming a phylogenetically unique clade that warrants further investigation.

Efficient CO_2_ utilization requires the presence of a support activase. The presence of two RuBisCO activase copies were identified in the *C*. *tobin* genome (one nuclear and one chloroplast encoded). This observation was extended to show that all haptophytes, cryptophytes, and stramenopiles for which nuclear and chloroplast genomes are available, have an identical coding profile for these activases. The requirement for exogenous B_12_ acquisition demonstrates co-dependence of this organism on eco-cohorts for survival.

In summary, the *C*. *tobin* genome has provided a wealth of new information. Observations reveal products that may be potentially useful in therapeutic application (e.g., xanthorhodopsins, polyketides) as well as data that may be of high value to algal commercialization and genetic engineering efforts.

## Materials and Methods

### Culture maintenance

*Chrysochromulina tobin* strain CCMP291, acquired from The National Center for Marine Algae by the Cattolico laboratory in 2006, was designated as P3. These cultures were maintained in 250 mL Erlenmeyer flasks containing 100 mL of RAC-1, a proprietary fresh water medium. Flasks were plugged with silicone sponge stoppers (Bellco Glass, Vineland, NJ) and capped with a sterilizer bag (Propper Manufacturing, Long Island City, NY). Large volume experimental cultures for genomic DNA and transcriptomic RNA harvesting were maintained in 1.0 L of RAC-1 medium that was contained in 2.8 L large-mouth Fernbach flasks. These flasks were plugged with hand-rolled, #50 cheese cloth-covered cotton stoppers and covered with a #2 size Kraft bag (Paper Mart, Orange, CA). All cultures were maintained at 20°C on a 12 hour light: 12 hour dark photoperiod under 100 μEm^-2^s^-1^ light intensity using full spectrum T12 fluorescent light bulbs (Philips Electronics, Stamford, CT). No CO_2_ was provided and cultures were not agitated.

Algal cultures were treated in the following manner to minimize bacterial contamination. P3 cultures were subject to re-iterative cell sorting using flow cytometry. *C*. *tobin* cells were stained for identification using BODIPY 505/515 (4,4-difluoro-1,3,5,7-tetramethyl-4-bora-3a,4a-diaza-s-indacene; Invitrogen, Carlsbad, CA), a neutral lipid binding fluorophore. Approximately 10 stained cells were sorted into a single well of a 96 well plate containing 100 μL RAC-1 medium and then transferred to 10 mL of RAC-1 medium in 50 mL plastic tissue culture flasks (Nunc, Roskilde, Denmark). This cell sorting process was carried out 4 times with the resulting culture being designated as P4. Cells obtained from reiterative flow cytometric selection (P4) were then treated in RAC-1 medium that contained either streptomycin (resulting in culture P5.5) or hygromycin (P5.6). Treatment with these two antibiotics was identical. Cells were exposed to a final concentration of 400 μg/mL antibiotic for 18 hours before 5 mL of treated cultures were transferred to 100 mL of antibiotic free RAC-1 medium. Cultures P5.5 and P5.6 were periodically tested for bacterial contamination using liquid LB medium made with RAC-1 medium in replacement of water. Sequencing data and recovery of a cultured bacterial isolate has shown that a single bacterial contaminant is still present in the P5.5 culture.

### Genomic DNA isolation

Total genomic DNA was collected from each of the P5.5 and P5.6 cultures using the Qiagen Genomic-tip Maxi DNA extraction protocol (Germantown, MD) with the following changes to the standard protocol. 1.5 x 10^8^ cells were harvested by centrifugation at 5,378 x g for 20 minutes and resuspended in lysis buffer (20 mM EDTA, pH 8.0; 10 mM Tris-base, pH 8.0; 1% Triton X; 500 mM guanidine; 200 mM NaCl). After 1.0 hour incubation at 37°C, RNase A was added to 200 μg/ml final concentration and the mixture incubated for 30 minutes at 37°C. Following the addition of 600 μL Proteinase K (20 mg/ml) (Sigma-Aldrich) incubation was continued at 50°C for 2.0 hours, mixing every 30 minutes by swirling. DNA preparation was transferred into a Qiagen DNA binding tip (Maxi size) that was equilibrated using the manufacturer’s instructions, and allowed to pass through the tip by gravity, while maintained at room temperature. The tip was then washed twice using Qiagen buffer QC. Fifteen mL of Buffer QF (at 37°C) was added to the tip to elute the DNA. DNA was precipitated by adding 10.5 mL of 100% room temperature isopropanol followed by centrifugation at 11,220 x g for 20 min at 4°C. The pellet was washed in 4 mL of 4°C 70% ethanol and centrifuged again using the same conditions. The DNA pellet was air dried for 5 min and resuspended in warmed Qiagen buffer EB (50°C) and incubated at 50°C for 2.0 hours. DNA solution was quantitated using a spectrophotometer and subsequently transferred to 1.7 mL Eppendorf tubes and stored at -80°C.

### Genome sequencing, assembly and annotation

The *C*. *tobin* genome was sequenced using a combination of Illumina and 454 sequencing. For Illumina, two shotgun libraries (2 X 100 and 1 x 150 base pair) were prepared using standard TruSeq protocols and sequenced on an Illumina HiSeq2000. Using the 454 Titanium platform, shotgun single-end and paired-end (10 kb insert) DNA libraries were prepared generating 4.7 million reads in total. The 454 single end and paired end data (insert size 8180 +/- 1495 bp) were assembled using Newbler, version 2.3 (release 091027_1459) (Roche). The sequences generated by the Illumina platform were assembled separately with VELVET, version 1.0.13 [95]. Consensus sequences from the VELVET and Newbler assemblies were computationally shredded into 10 kb fragments and were re-assembled with reads from the 454 paired end library using parallel‬ Phrap, version 1.080812 (High Performance Software, LLC).

The final draft genome assembly produced over three thousand contigs. Gene annotation was carried out using the MAKER2 training and annotation pipeline [96]. After masking repeated genomic elements using Repeatmasker [97], genes were modeled by combining several methods in MAKER2: a) aligning *C*. *tobin* transcriptomic BLASTn hits as EST evidence; b) aligning to *Emiliania huxleyi* ESTs using tBLASTx; c) using the assembled RNAseq data for gene prediction with Tophat [98] and Cufflinks [99]; d) aligning all CEGMA (Core eukaryotic genes) [100] genes to the *C*. *tobin* contigs using BLASTx; e) Augustus [101] for *ab initio* models trained on the gene structures of *Chlamydomonas reinhardtii*; f) SNAP [102] for *ab initio* models trained on Hidden Markov Models (HMMs) of the predicted genes by cufflinks and Tophat models; g) GenemarkES for *ab initio* gene models [103]. A total of 16,777 genes were annotated using the above method. Of these, 10,293 were supported by BLAST homology using BLAST2GO and 6,484 are considered novel genes.

### Functional annotation

BLAST2GO [104] was used to attach functional annotation to gene call predictions. First, BLASTp was used to search the non-redundant protein database (nr) with a Blast Expect Value cutoff of 1e^-6^. Blast2Go Mapping was performed followed by Annotation using E-Value-Hit-Filter: 1e^-6^, Annotation cutoff of 55 and GO weight of 5. These gene annotations were used in the remainder of the gene analyses unless the manual curation of a gene gave evidence supporting a manual annotation.

### RNA sequencing

Twelve 1 L cultures were seeded at a starting density of 50,000 cells/ mL, 66 hours prior to the first harvesting time point (Dark hour 6) using inoculation cultures that were maintained for 7 days at standard conditions (see above). Total RNA was purified using a modified TRIzol preparation: 1.5 x 10^8^ cells were collected per RNA isolation sample. *C*. *tobin* cells were centrifuged at 8,600 x g for 20 minutes in 500 mL polypropylene centrifuge bottles. The supernatant was decanted and 5 mL of TRIZOL reagent (Invitrogen) was added to the cell pellet. Cells were resuspended by pipetting and vortexing for 1 minute. The homogenate was transferred equally into four microcentrifuge tubes. To each tube, 250 μL of chloroform was added. Each tube was shaken by hand vigorously for 15 seconds and subsequently centrifuged for 15 minutes at 12,000 x g at 4°C. The mixing and centrifugation was repeated once. After the second centrifugation, the top aqueous phase was transferred to a new microcentrifuge tube being sure not to disturb the lower phenol/chloroform phase. Ice cold isopropanol (625 μL) was added to each of the 4 tubes containing the aqueous phase and incubated at -20°C overnight. The samples were then centrifuged at 12,000 x g for 10 minutes at 4°C. The supernatant was removed and the pellet washed with 1.25 mL of 75% ice cold ethanol followed by a 5 minute centrifugation at 7,400 x g. The ethanol wash and centrifugation step was repeated one time. The pellet was dried for 10 minutes and resuspended in 30 μL RNase free water (Qiagen). Four samples were combined into a single tube and treated with RNase free DNase for 90 minutes at 37°C. Samples were then cleaned using a Qiagen RNeasy MinElute clean up protocol as specified by the manufacturer’s instructions. Samples were stored at -80°C

Poly-A selection was carried out followed by library preparation using a TruSeq library kit (Illumina). Sequencing was done on the Illumina high-seq and generated 100 bp paired reads. For each time point collected, 15–30 million reads were generated. Reads were trimmed and groomed [105]. Tophat version 1.5 [98] was used to assemble the sequences using the *C*. *tobin* draft genome as a reference. Cufflinks (v2.1.1) was used to estimate FPKM (fragments per kilobase of exon per million mapped reads) for each transcript at each time point [106]. To determine which subset of the transcriptomic data to include in the global analysis a high expression and high variance selection method was implemented to determine genes that were 1) highly expressed and 2) had great differences in expression between 2 or more time points. This gene selection was done using an in-house derived formula (“MeanNeighbor”) that takes each time point and compares the expression level (FPKM value) average across adjacent time points. This method was implemented in R using the following function: MeanNeighbor = function(x) {mean(abs(x[2:length(x)]-x[1:length(x)-1)]))}. The top 1000 genes as scored by MeanNeighbor value were then plotted as a heatmap using a normalization constant so that all values of gene expression FPKM could be represented by a relative level between -3 and +3. First, each individual FPKM value was subtracted from each transcript’s average FPKM value of all 7 time points. The resulting value at each time point was then divided by the standard deviation, giving a relative expression level to be used in the generation of the heatmap. The global heatmap was generated using the R library “pheatmap” [107] using the “ward” clustering method. Fisher’s exact test was used in Blast2GO to determine GO term overrepresentation in each group based on the annotation of GO terms by Blast2GO. The p-value cutoff for this was set at 0.05. For group 3, over 100 members were obtained so the p-value cutoff was lowered to 1e^-7^ to generate the graphs used in Fig 4.

RNA seq data was also assembled *de novo* to identify genes that may have not been present or were mis-assembled in the final *C*. *tobin* nuclear genome draft. Trinity [108] was used to assemble transcripts, which were used to create an additional BLAST database used in identifying NAD genes, cobalamin synthesis, and polyketide related genes in addition to those found in the nuclear genome draft.

### Lipid measurements

#### Flow cytometry

Total cellular neutral lipid content of the experimental cultures was measured as follows. The BODIPY 505/515 (Invitrogen, Eugene, OR) stock solution was prepared by adding the dry BODIPY 505/515 powder to 99% pure DMSO for a 5 mM final stock concentration. 7.5 μL of the stock solution was diluted 3:1 in 22.5 μL of RAC-1 medium for the working stock solution. A 990 μL aliquot of cell culture and 10 μL of working stock solution were placed into a 12 x 75 mm glass tissue culture tube for use in flow cytometric measurements. The tube was capped, inverted to mix the dye, and incubated in the dark at room temperature for at least one minute. BODIPY 505/515 labeled samples were measured using a BD Accuri C6 flow cytometer in the FL1 channel (excitation: 488 nm; 530/30 nm emission). Unstained cells were used as a control. The BODIPY 505/515 background was negligible. Because BODIPY 505/515 stained samples are spectrally distant and of much higher signal strength than chlorophyll auto-fluorescence and cellular debris, experimental samples are easily gated.

#### Gas chromatography/mass spectrometry (GC/MS)

Samples were collected for total fatty acid analysis when algal cultures were in stationary growth phase (S4 Table). GC/MS analysis was performed using the sub-microscale in-situ method devised in this laboratory [41]. Briefly, 10 mL culture aliquots (quadruplicate samples) were placed in new 10 mL Pyrex glass tubes (Fisher Scientific, Pittsburgh, PA), centrifuged at 5,900 x g for 20 min at 4°C, and the pelleted cells flash-frozen in liquid nitrogen. Samples were then stored at -80°C before lyophilization and chemical processing. The fatty acids present in the lyophilized samples were transmethylated to fatty acid methyl esters in-situ, catalyzed by boron trifluoride in methanol. A two-component triglyceride surrogate was added to the sample prior to transmethylation to account for any variation in methylation or sample handling prior to internal standardization. After transmethylation, the analytes were separated from the other compounds present in the sample using a two-phase (brine and isooctane), two-step phase separation. An internal standard of deuterated aromatics was then added to the sample. Analyte separation and detection was performed using GC/MS. Quantitation was performed against a 27-component external standard.

#### Fluorescein diacetate flow cytometry assay

The fluorescein diacetate (FDA) assay used in this study was modified from a study by Jochem [109]. A 5 mg/mL stock solution of FDA, 3,6-Diacetoxyfluoran, Di-O-acetylfluorescein (Sigma-Aldrich, St. Louis, MO) was prepared in 99.9% anhydrous DMSO (Sigma-Aldrich, St. Louis, MO). This stock solution was stored in a 15 mL Falcon tube at 4°C. For experimental runs, the stock solution was thawed and diluted 100-fold with chilled double-distilled water to make the 50 μg/mL FDA working solution. 330 μL of the working solution was added to 10 mL of cell culture in a 12 x 75 mm glass tissue culture tube (BD Biosciences, San Jose, CA, USA). After gentle vortexing for approximately 5–10 seconds on a low setting, 1.0 mL of each cell/FDA sample was placed into 8 replicate wells of a clear 96-well plate (BD Biosciences, San Jose, CA, USA). The plate was incubated for 10 minutes at 20°C under normal room illumination or shielded from all light depending on the hour of the light:dark cycle. The FDA signal was measured using an Accuri C6 flow cytometer in the FL1 channel (excitation: 488 nm; emission: 530/30 nm). Unstained cells were used as a control.

#### Modeling of xanthorhodopsin

The Rosetta comparative modeling protocol [110] was used to model the tertiary structure of *C*. *tobin* xanthorhodopsin using the template 3DDL [75] from the protein data bank. Approximately 41,000 trajectories were performed using Rosetta version 3 [111] with a new energy function [112]. Secondary structure prediction for the query sequence was made using PsiPred [113] and fragments (3- and 9- residue long) were created using Robetta server [114]. The models were clustered based on their backbone RMSD and top representatives based on lowest full atom Rosetta energy and were visually evaluated using protein structure visualization software, PyMOL (v0.99, Schrödinger, LLC). Top 10 models by total score (-404 to -400 Rosetta Energy Units or -1.5 REU/residue) out of approximately 41,000 generated models were within around 2 Å Cα-RMSD from the template (3DDL). The seven helices of the model aligned well (Fig 9) with maximum deviation in the loop region connecting second and third helix of the model (residues 71–93).

Docking studies of retinal in the putative pocket of *C*. *tobin* xanthorhodopsin structural models were also performed. Retinal molecule coordinates were taken from the structural template 3DDL. Carbonyl oxygen and protons were added to the molecule using Avogadro molecule editor software [115]. The five rotatable bonds in a retinal molecule were sampled at two more states, ±30° from the dihedral angles that were observed in the structure, 3DDL. Approximately 200 conformers of retinal molecule were generated that had intra-molecule full atom repulsive energy, as calculated by Rosetta [116], not worse than the starting molecule used for conformer generation. Random docking of retinal conformers in top 10 selected comparative models of *C*. *tobin* xanthorhodopsin was achieved by the RosettaLigand protocol [117]. A total of 6,300 dock trajectories were run for each comparative model and filtered based on ligand binding energy and ligand RMSD from the ligand conformation in the template (3DDL).

#### Phylogenetic analysis

NCBI non-redundant (nr) and MMETSP [3] databases were mined for CbbX, rhodopsin, and *psbA* sequences for phylogenetic analysis. Relevant CbbX (262 sequences), rhodopsin (81 sequences), and *psbA* (18 taxa) sequences were aligned in MUSCLE [118] and manually trimmed to leave 257 amino acid, 231 amino acid, and 857 nucleotide alignments, respectively. Best choice protein and nucleotide models were queried using ProtTest 2.4 [119] and jModelTest [120], respectively. The WAG+I+G model suited both the CbbX and rhodopsin datasets, and the *psbA* data were best modeled under GTR+I+G. The CbbX, rhodopsin and *psbA* gene trees were generated using the online CIPRES Science Gateway (www.phylo.org) with MrBayes [121] using the following conditions: 2 runs of four chains, with 3 million, 5 million, or 500,000 generations, respectively, and 25% burn-in. Convergence of the Bayesian analysis was viewed with Tracer [122]. Maximum-likelihood analyses were performed using RAxML [123], also in the CIPRES Science Gateway. The resulting output was visualized in Figtree v1.4.2 (http://tree.bio.ed.ac.uk/software/figtree).

## Supporting Information

S1 Dataset Complete gene expression data set (FPKM values at 7 time points). (XLSX)

S2 Dataset Complete list of GO terms and genes in each of the 7 groups defined by gene expression patterns. (XLSX)

S3 Dataset Detailed fatty acid profile data for each pie chart displayed in Fig 7. (XLS)

S4 Dataset Nexus alignment and sequences of *psb*A found in the phylogeny in Fig 7. (NEX)

S5 Dataset Nexus alignment, sequences and.tre files of the rhodopsin phylogeny found in Fig 10. (ZIP)

S6 Dataset Nexus alignment, sequences and.tre files of the CbbX phylogeny found in Fig 11. (ZIP)

S1 Fig Cumulative distribution function (CDF) plots of mRNA and CDS size. (TIF)

S2 Fig Transcription of genes important for fatty acid chain modification. (A) Fatty acid elongase transcript expression and (B) high and low levels of desaturase transcript expression.
(TIF)

S3 Fig Change in triacylglycerol lipase transcript abundance over a 24 hour light/dark photoperiod. (TIF)

S4 Fig Expression of esterase lipase transcripts over 7 time points. (TIF)

S5 Fig Functionally important amino acids of erythromycin esterase are completely conserved in identity, including H46/56 (*Massillia* sp. JS1662 numbering/*C*. *tobin*) that is indispensable for catalytic function, and the 9 amino acids (E43/53; T45/55; H46/56; G143/193; D145/194; 261/315; H290/345; N291/346; H293/348) (red highlight) that are critical for maintaining the active site pocket of the enzyme. (TIF)

S6 Fig Transcript abundance of *C*. *tobin* xanthorhodopsin. The single *C*. *tobin* xanthorhodopsin gene is highly and temporally expressed.
(TIF)

S7 Fig Transcript abundance of both *C*. *tobin bat* genes. (TIF)

S1 Table Gene calling and annotation statistics. (PDF)

S2 Table Select haptophyte and stramenopile genome sizes, predicted genes and protein coding sequences. (PDF)

S3 Table Type III Polyketide synthase genes. The *C*. *tobin* genome and transcriptome provide evidence of smaller, single function, Type III PKSs or remnants of Type I PKS domains.
(PDF)

S4 Table Summary of each organism represented in the fatty acid content analysis. (PDF)
