# Supplementary figures and images for: Genome Sequence and Transcriptome Analyses of Chrysochromulina tobin: Metabolic Tools for Enhanced Algal Fitness in the Prominent Order Prymnesiales (Haptophyceae)
Source: PLoS Genet. 2015 Sep 23;11(9):e1005469. doi: 10.1371/journal.pgen.1005469 (PMC4580454; doi:10.1371/journal.pgen.1005469)

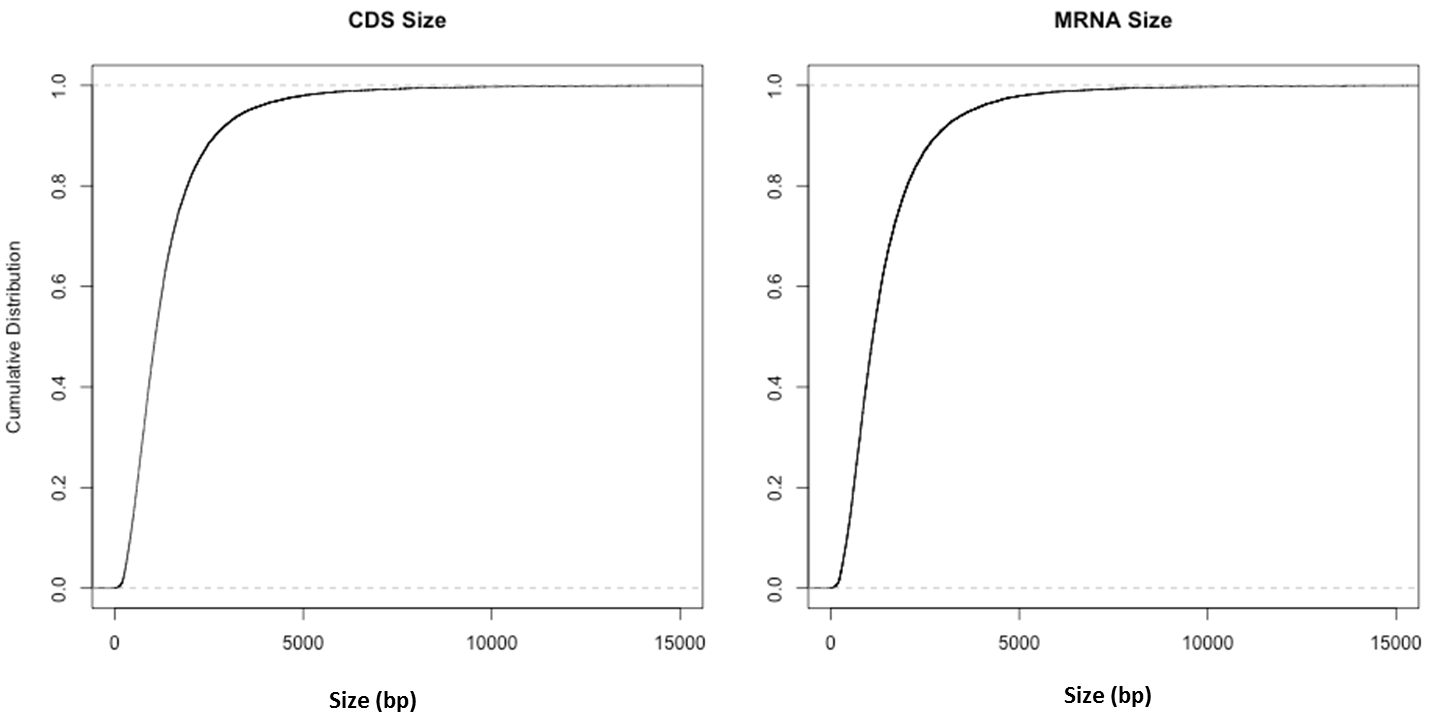

Supplement: S1 Fig — (TIF) [file pgen.1005469.s007.tif]

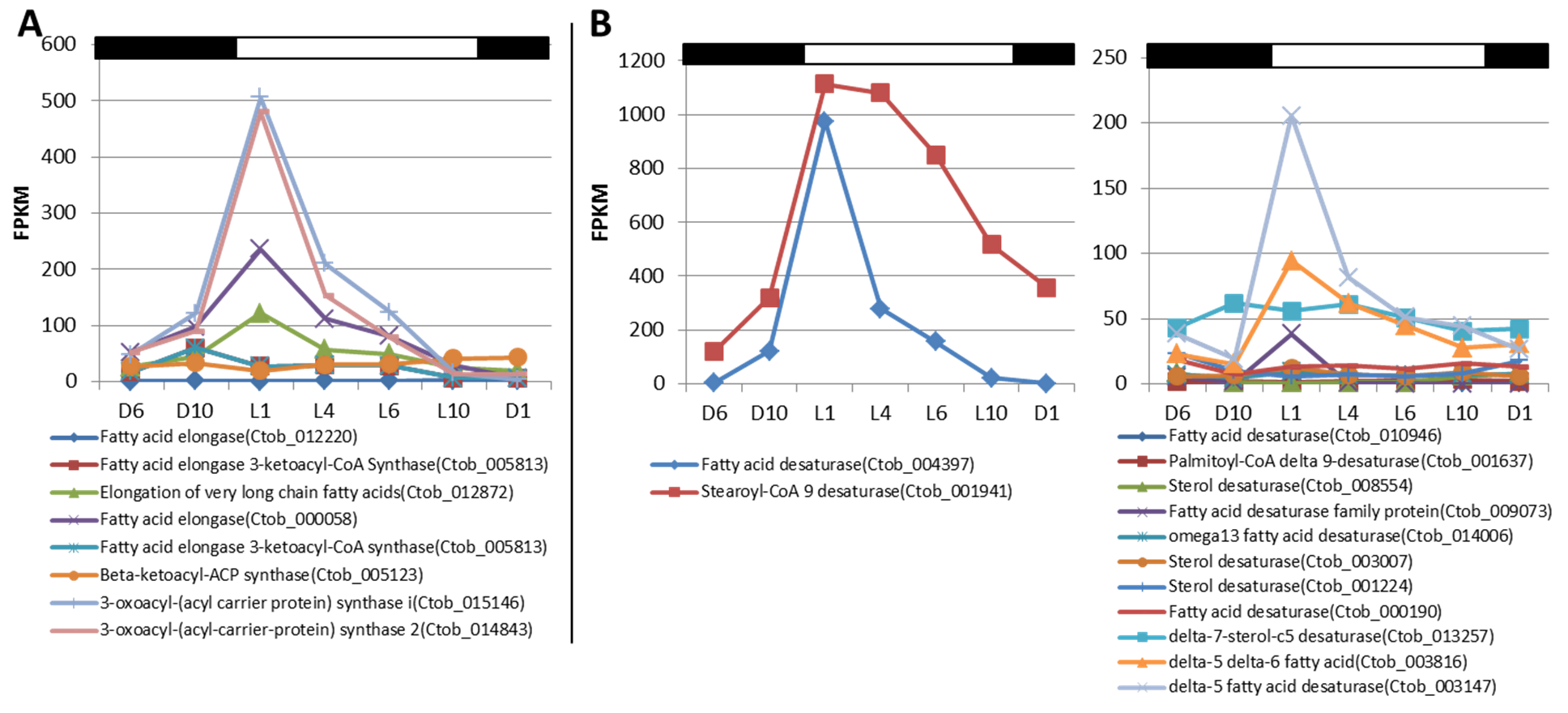

Supplement: S2 Fig — (A) Fatty acid elongase transcript expression and (B) high and low levels of desaturase transcript expression. (TIF) [file pgen.1005469.s008.tif]

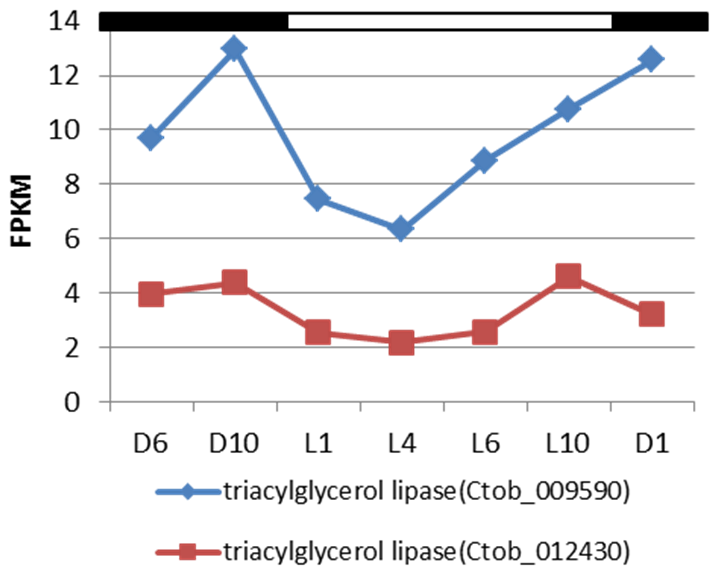

Supplement: S3 Fig — (TIF) [file pgen.1005469.s009.tif]

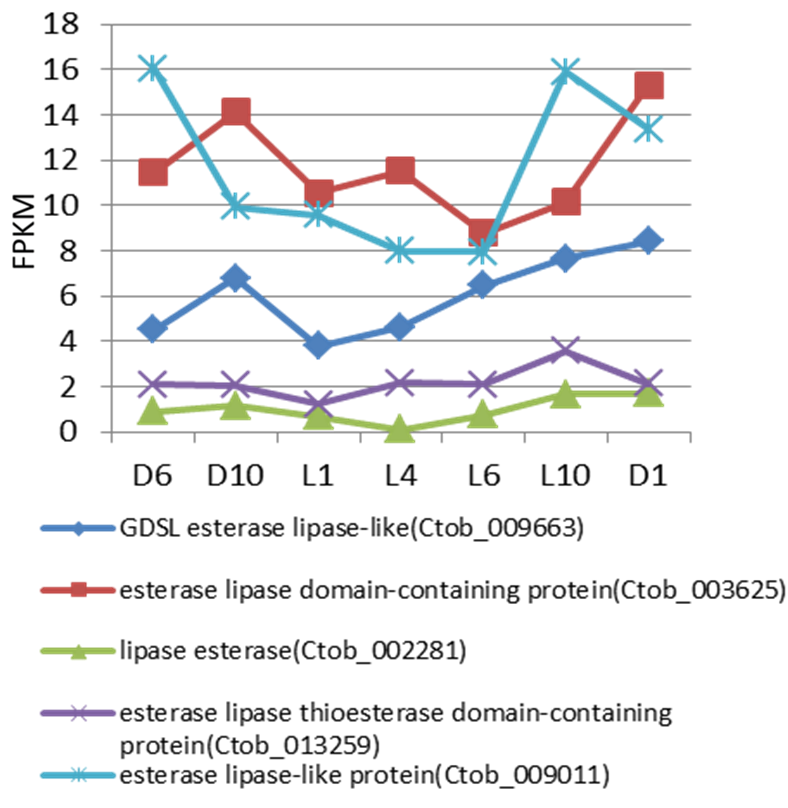

Supplement: S4 Fig — (TIF) [file pgen.1005469.s010.tif]

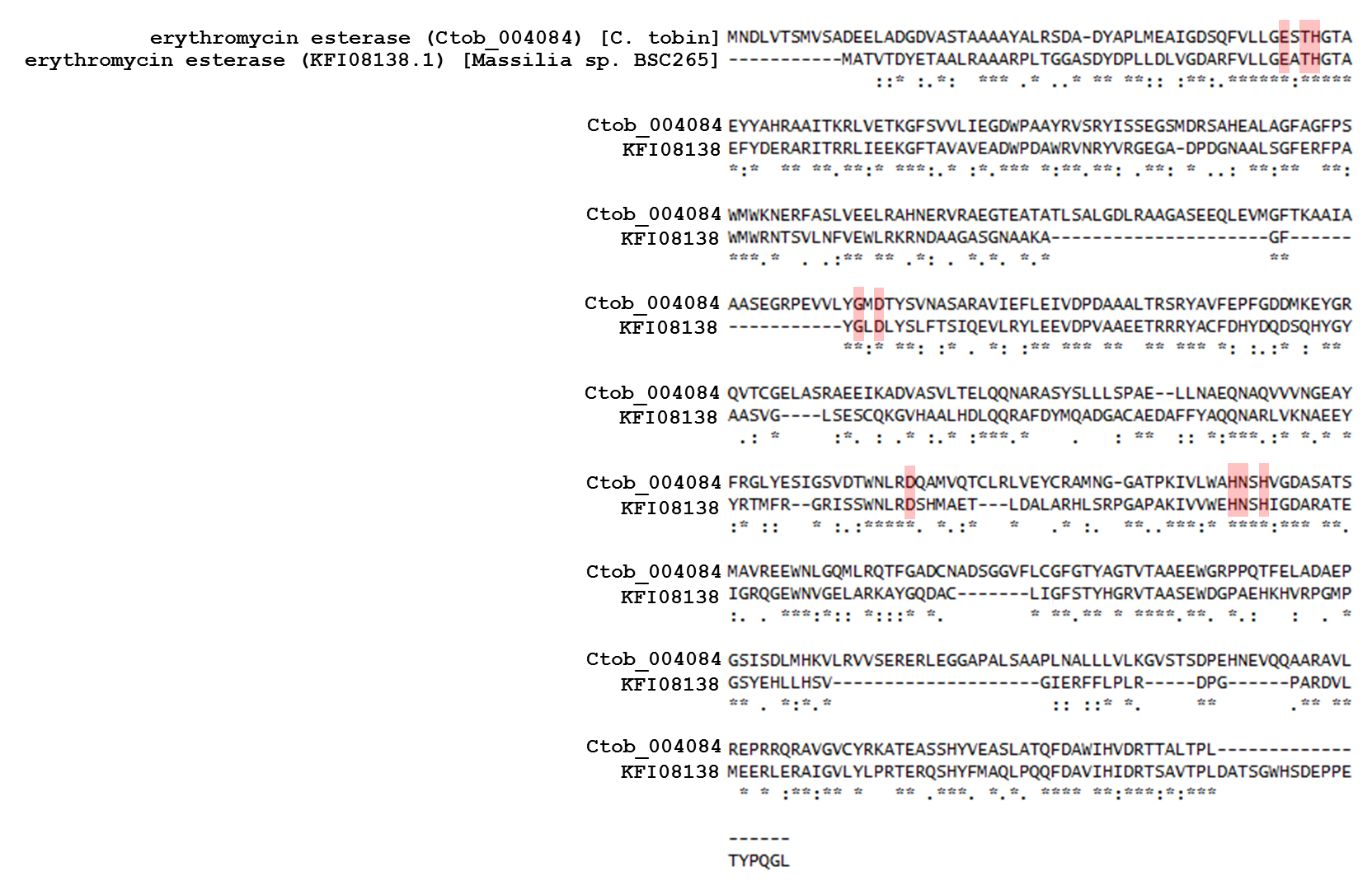

Supplement: S5 Fig — (TIF) [file pgen.1005469.s011.tif]

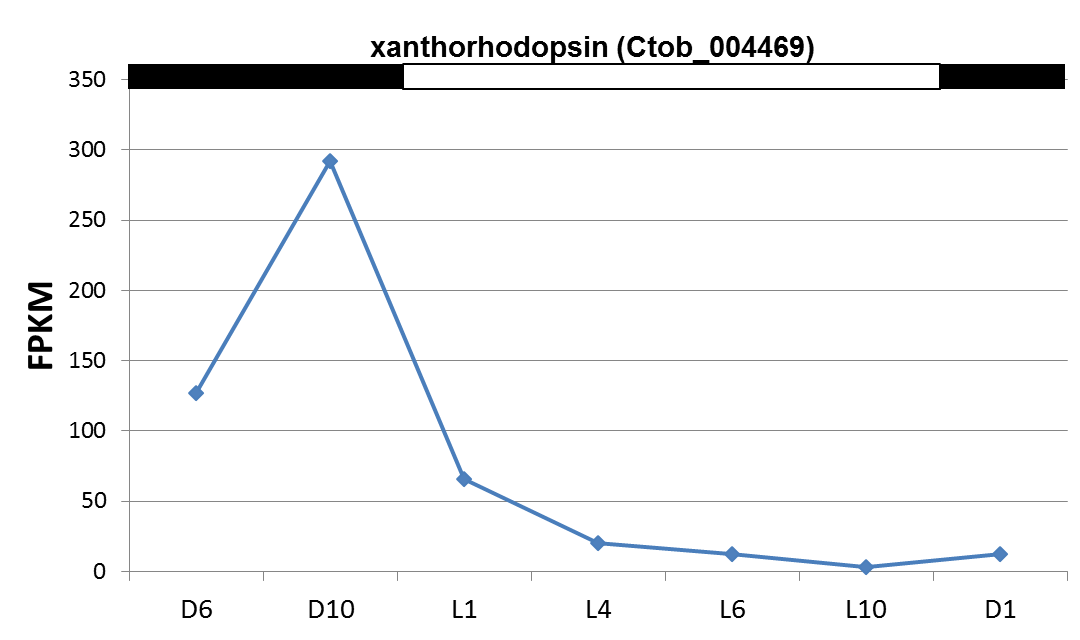

Supplement: S6 Fig — The single C. tobin xanthorhodopsin gene is highly and temporally expressed. (TIF) [file pgen.1005469.s012.tif]

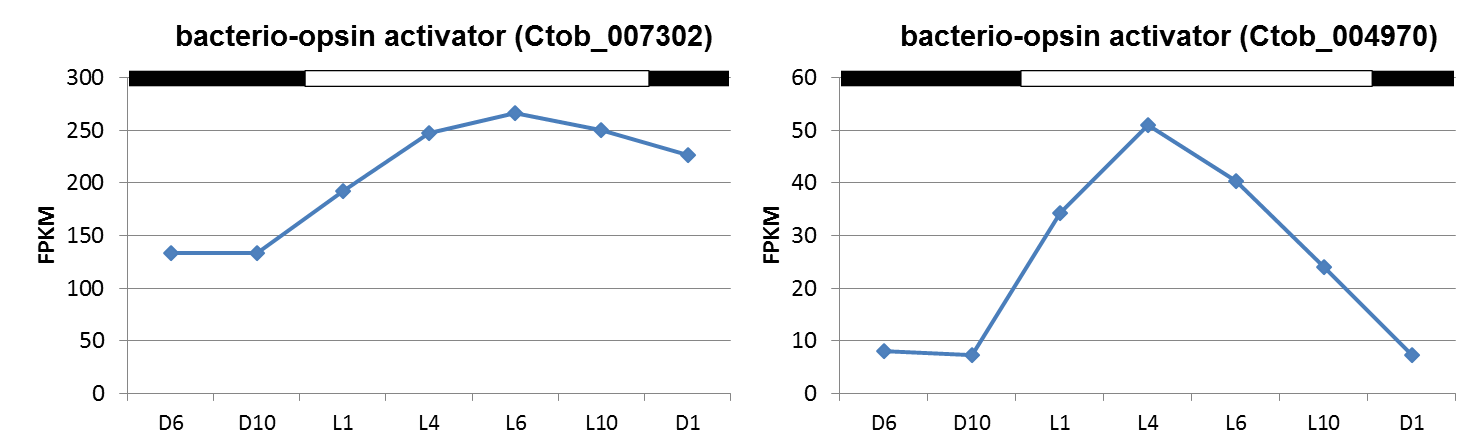

Supplement: S7 Fig — (TIF) [file pgen.1005469.s013.tif]
