# Supplementary material for: Genome Sequence and Transcriptome Analyses of Chrysochromulina tobin: Metabolic Tools for Enhanced Algal Fitness in the Prominent Order Prymnesiales (Haptophyceae)
Source: PLoS Genet. 2015 Sep 23;11(9):e1005469. doi: 10.1371/journal.pgen.1005469 (PMC4580454; doi:10.1371/journal.pgen.1005469)
Supplement: S1 Table — (PDF) [file pgen.1005469.s014.pdf]

Supplementary Table 1: Gene calling and annotation statistics for the *Chrysochromulina tobin* genome

Protein coding genes (nuclear genome)	16777
Genes supported by BLAST homology (Blast expect value < $1e^{-6}$)	10293 (61.4%)
Genes not supported by BLAST homology	6484 (38.6%)
Average gene length	1899 bp
Average CDS length	1405 bp
Average exon length	445 bp
Average exons per gene	2.28
Average intron length	297 bp
Average introns per gene	1.28
