# Supplementary material for: Genome Sequence and Transcriptome Analyses of Chrysochromulina tobin: Metabolic Tools for Enhanced Algal Fitness in the Prominent Order Prymnesiales (Haptophyceae)
Source: PLoS Genet. 2015 Sep 23;11(9):e1005469. doi: 10.1371/journal.pgen.1005469 (PMC4580454; doi:10.1371/journal.pgen.1005469)
Supplement: S2 Table — (PDF) [file pgen.1005469.s015.pdf]

Alga	Genome Size (Mb)	Predicted Genes	% protein coding	Reference
Haptophytes				
Chrysochromulina tobin	59	16777	40.4	This paper
Emiliania huxleyi	142	30569	21.6	Read BA, Kegel J, Klute MJ, Kuo A, Lefebvre SC, Maumus F, et al. Pan genome of the phytoplankton *Emiliania* underpins its global distribution. Nature. 2013;499: 209–213. doi:10.1038/nature12221
Stramenopiles				
Aureococcus anophagefferens	56.7	11500	42.0	Gobler CJ, Berry DL, Dyhrman ST, Wilhelm SW, Salamov A, Lobanov AV, et al. Niche of harmful alga *Aureococcus anophagefferens* revealed through ecogenomics. Proc Natl Acad Sci U S A. 2011;108: 4352–4357. doi:10.1073/pnas.1016106108
Ectocarpus siliculosus	214	16256	16.0	Cock JM, Sterck L, Rouzé P, Scornet D, Allen AE, Amoutzias G, et al. The *Ectocarpus* genome and the independent evolution of multicellularity in brown algae. Nature. 2010;465: 617–621. doi:10.1038/nature09016
Nannochloropsis oceanica	30.1	9915	52.1	Wang D, Ning K, Li J, Hu J, Han D, Wang H, et al. *Nannochloropsis* genomes reveal evolution of microalgal oleaginous traits. PLoS Genet. 2014;10: e1004094. doi:10.1371/journal.pgen.1004094
Phaeodactylum tricornutum	27	10681	57.3	Bowler C, Allen AE, Badger JH, et. al. The Phaeodactylum genome reveals the evolutionary history of diatom genomes. Nature. 2008 Nov 13;456(7219):239-44. doi:10.1038/nature07410
Thalassiosira pseudonana	34.5	11242	32.7	Armbrust EV, Berges JA, Bowler C, Green BR, Martinez D, Putnam NH, et al. The genome of the diatom *Thalassiosira pseudonana* : ecology, evolution, and metabolism. Science. 2004;306: 79–86. doi:10.1126/science.1101156
Phytophthora infestans	228.5	18179	9.9	*Phytophthora infestans* Sequencing Project, Broad Institute of Harvard and MIT (http://www.broadinstitute.org/)
