# Supplementary material for: Genome Sequence and Transcriptome Analyses of Chrysochromulina tobin: Metabolic Tools for Enhanced Algal Fitness in the Prominent Order Prymnesiales (Haptophyceae)
Source: PLoS Genet. 2015 Sep 23;11(9):e1005469. doi: 10.1371/journal.pgen.1005469 (PMC4580454; doi:10.1371/journal.pgen.1005469)
Supplement: S3 Table — The C. tobin genome and transcriptome provide evidence of smaller, single function, Type III PKSs or remnants of Type I PKS domains. (PDF) [file pgen.1005469.s016.pdf]

Species	Culture Collection ID	Growth Media*	Temperature (°C)	Light intensity ($\mu Em^{-2}s^{-1}$)
Prymnesium parvum	CCMP 3037	COREs/FSW[A]	20	30
Prymnesium parvum	CCMP 709	COREs/FSW	15	20
Pavlova lutheri	UWCC MI 631	COREs[B]	10	20
Chrysochromulina kappa	CCMP 288	COREs/FSW	20	40
Phaeocystis globosa	CCMP 2754	COREs/FSW	20	40
Emiliania huxleyi	CCMP 1742	L1 no Si[C]	20	35
Cryptomonas sp.	UWCC FW 704	COREs	15	20
Cruciplacolithus neohelis	UWCC MI 693	COREs	20	30
Isochrysis galbana	UWCC MI 635	f/2 no Si[D]	10	20
Phaeocystis globosa	CCMP 627	L1 no Si	20	40
Chrysochromulina ericina	CCMP 282	COREs/FSW	20	40
Chrysochromulina polylepis	CCMP 286	L1 no Si	15	20
Chrysochromulina parva Lackey	CCMP 291	COREs	15	30
Hymenomonas sp.	UWCC MI 398	COREs	15	20
Prymnesium parvum	UWCC MI 325	COREs/FSW	10	20
Rhodomonas sp.	CCMP 318	O-3[E]	15	20

*Media (A) and (B) are proprietary. Media (C) and (D) are availible at https://ncma.bigelow.org.ecology
Media (E) is found in M.B. Lakeman, P. von Dassow, R.A. Cattolico, The strain concept in phytoplankton, Harmful Algae 8 (5) (2009) 746–758.
