# Supplementary material for: Genome Sequence and Transcriptome Analyses of Chrysochromulina tobin: Metabolic Tools for Enhanced Algal Fitness in the Prominent Order Prymnesiales (Haptophyceae)
Source: PLoS Genet. 2015 Sep 23;11(9):e1005469. doi: 10.1371/journal.pgen.1005469 (PMC4580454; doi:10.1371/journal.pgen.1005469)
Supplement: S4 Table — (PDF) [file pgen.1005469.s017.pdf]

PKS gene	Average expression level (FPKM)	Contig	Notes
Ctob_016596	0.57	Contig7855	Adjacent to this gene, in contig 7855, are unannotated ketoreductase and fatty acid synthase complexes – key components of polyketide synthases. Contig 7855 is only 2746 bp in length.
Ctob_009309	3.6	Contig8049	Truncated PKS C-terminus
Ctob_011429	38.2	Contig8528	
